# The role of taurine in male reproduction: Physiology, pathology and toxicology

**DOI:** 10.3389/fendo.2023.1017886

**Published:** 2023-01-18

**Authors:** Yuanyuan Li, Qianwen Peng, Jia Shang, Wanglin Dong, Sijia Wu, Xiajun Guo, Zhenxing Xie, Chaoran Chen

**Affiliations:** ^1^ Institute of Nursing and Health, School of Nursing and Health, Henan University, Kaifeng, Henan, China; ^2^ Arts Department, School of Kaifeng Culture and Tourism, Henan, Kaifeng, China; ^3^ School of Basic Medical Science, Henan University, Henan, Kaifeng, China

**Keywords:** male reproduction, taurine, antioxidant, testis, sperm

## Abstract

Taurine, a sulfur-containing amino acid, has a wide range of biological effects, such as bile salt formation, osmotic regulation, oxidative stress inhibition, immunomodulation and neuromodulation. Taurine has been proved to be synthesized and abundant in male reproductive organs. Recently, accumulating data showed that taurine has a potential protective effect on reproductive function of male animals. In physiology, taurine can promote the endocrine function of the hypothalamus-pituitary-testis (HPT) axis, testicular tissue development, spermatogenesis and maturation, delay the aging of testicular structure and function, maintain the homeostasis of the testicular environment, and enhance sexual ability. In pathology, taurine supplement may be beneficial to alleviate pathological damage of male reproductive system, including oxidative damage of sperm preservation *in vitro*, testicular reperfusion injury and diabetes -induced reproductive complications. In addition, taurine acts as a protective agent against toxic damage to the male reproductive system by exogenous substances (e.g., therapeutic drugs, environmental pollutants, radiation). Related mechanisms include reduced oxidative stress, increased antioxidant capacity, inhibited inflammation and apoptosis, restored the secretory activity of the HPT axis, reduced chromosomal variation, enhanced sperm mitochondrial energy metabolism, cell membrane stabilization effect, etc. Therefore, this article reviewed the protective effect of taurine on male reproductive function and its detailed mechanism, in order to provide reference for further research and clinical application.

## 1 Introduction

Taurine is a sulfur-containing nonprotein amino acid that has been found to be one of the most abundant amino acids in mammalian plasma and tissues. Although taurine is not involved in the synthesis and metabolism of protein, as a functional component, it is involved in a variety of physiological functions, including bile formation in the liver, modulation of calcium flow, osmoregulation, neurotransmitter or neuromodulator, antiarrhythmic activity, etc. (1). The sources of taurine in the body are biosynthesis and dietary intake. Taurine is mainly synthesized by methionine and cysteine in liver, and its synthesis ability is limited (2). On the other hand, dietary taurine is mainly obtained from meat, seafood, or energy drinks (3, 4). In addition, dietary taurine deficiency in species with low taurine biosynthesis rate (such as cats and foxes) can lead to many diseases, such as retinal degeneration (5), dilated cardiomyopathy (6), immune dysfunction (7) and reproductive defects (8). Therefore, taurine treatment is beneficial to various pathologies.

Taurine has been proved to be biosynthesized in the reproductive system of male animals. Cysteine sulfinate decarboxylase (CSD) is a key enzyme in taurine biosynthesis pathway (9). Studies have shown that CSD mRNA and protein are expressed in vas deferens, epididymis and testis, especially in Leydig cells of testis (10). Taurine, as a simple but unique amino acid, has a wide range of physiological functions in the male reproductive system. First, taurine is concentrated in the mitochondria of various cells (11), which can be used as an antioxidant to prevent oxidative stress in testicular tissue by protecting mitochondrial structure and functional integrity (12), and improve the sperm viability and motility (13). Secondly, taurine may also act as a capacitating agent (14, 15), as well as a membrane-stabilized factor (16) and sperm motility factor (17). Additionally, further studies have found that taurine has protective effects on reproductive toxicity induced by heavy metals or some drugs (13, 18). These results indicated that taurine may be beneficial to the male reproductive system. Here, we review the role of taurine in maintaining physiological function of the male reproductive system, inhibiting pathological developments and alleviating toxic damage, hoping it provides ideas for future research.

## 2 The physiological role of taurine in male reproduction

### 2.1 Synthesis of taurine in testis

Taurine exists in high concentrations in reproductive tissues and interstitial fluids of both male and female mammals, such as uterine fluid (19) and oviduct (20, 21) of females, semens (22–24) and epididymal tissues (24, 25) of males. In male reproductive organs, taurine has been detected by immunohistochemistry in Leydig cells of the testis, vascular endothelial cells, and other Leydig cells, as well as epithelial cells of the efferent ducts (26). Semen is rich in taurine. Cumulative data show that taurine is much higher than other amino acids in semen of humans, hamsters, bulls, boars, dogs, pigs and guinea pigs (14, 23, 24, 27). Taurine is also abundant in human semen. Taurine content in human semen was reported to be from 319 to 1590 μmol/L and was maintained at a 10 times higher level than in the blood (27). Moreover, taurine content in human sperm ranged from 17nmol/mg DNA to 348 nmol/mg DNA, and taurine content ranged from 0nmol/mg DNA to 251 nmol/mg (27). Interestingly, the average content of hypotaurine in fertile men’s sperm was four times higher than that in infertile men, whereas the average content of taurine in fertile men’s sperm was lower than that in infertile men (28). Some researchers have speculated that the conversion of hypotaurine to taurine in sperm exposed to oxidative stress conditions may be an indicator of impaired sperm fertilization potential (29), thus the hypotaurine content in human sperm may be related to fertilization rates.

The expression of two key enzymes for taurine synthesis, such as CSD and CDO, has also been detected in male reproductive organs. For testis, previous studies have reported that the expression of CSD has been detected in the testis (30) and accessory gonads (31) such as epididymis, ductus deferens and anterior prostate of male animals. It was also found that inhibition of CSD mRNA expression in testicular interstitial cells significantly reduced T secretion (30). In addition, some researchers have proposed that there were species-specific differences in CSD activity, such as high levels of CSD activity in rats and dogs that rapidly synthesize taurine compared to cats, monkeys or humans (32), so species with lower CSD activity (e. g., cats) have a very limited ability to synthesize taurine from cysteine, and all essential amino acids (including taurine) must be provided in the diet (33). CDO is mainly expressed in caput epididymis and may be crucial for the synthesis of taurine in epididymis. Taurine concentrations in CDO^-/-^ sperm were found to be significantly decreased in the epididymal intracavity fluid and in the sperm cytoplasm (34). Further research shows that the progestin 17α, 20β-dihydroxy-4-pregnen-3-one (DHP) can regulate the synthesis of taurine in testis by promoting the expression of cysteine dioxygenase (CDO) mRNA in eel (35).

### 2.2 The physiological role of taurine in testis

**Figure 1:**
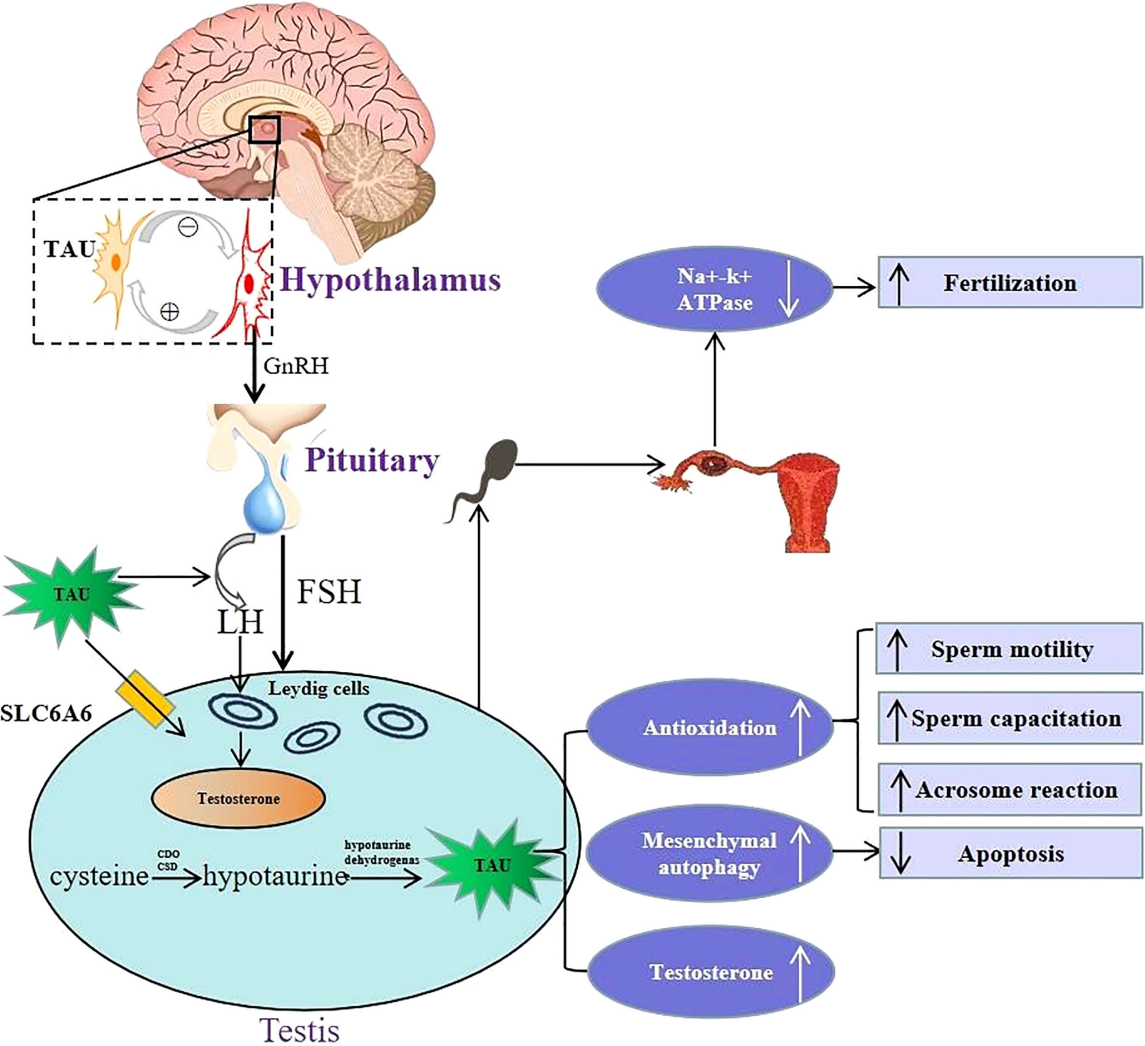
Schematic illustration of physiological role of taurine in hypothalamus-pituitary-testis endocrine axis. Top left portion shows the positive and negative feedback between taurine-secreting neurons and GnRH-secreting neurons in the hypothalamus. Lower left portion indicates that taurine regulates the secretion of FSH and LH by directly acting on the pituitary, and then LH acts on Leydig cells to promote testosterone secretion. Moreover, taurine can be transported to testis through transporter SLC6A6, and can also be synthesized endogenously in testis. Lower right portion indicates that the physiological mechanism and effects of taurine in testis, including: (i) enhancing antioxidation to promote spermatogenesis, sperm capacitation and acrosome reaction; (ii) enhancing the autophagy process of interstitial cells to inhibit cell apoptosis; (iii) promoting the secretion of testosterone. Top right portion shows in the fallopian tube, taurine reduced cellular K+ influx by inhibiting Na^+^–K^+^ ATPase activity, thereby avoiding high K+ inhibition of sperm motility to promote conception. TAU, taurine; SLC6A6, Sodium-and chloride-dependent taurine transporter; CDO, cysteine dioxygenase; CSD, cysteine sulfinate decarboxylase; GnRH, gonadotropin-releasing hormone; FSH, follicle-stimulating hormone; LH, luteinizing hormone.

The HPT axis is a key regulating system balancing male reproductive-endocrine function (36). Studies show that taurine can regulate male reproductive functions *via* acting on the HPT axis (shown in **Figure 1**). First, the effect of taurine on GnRH in hypothalamus is mainly stabilizing its basic secretion *via* negative feedback between neurons secreting the two substances (37). In the pituitary, it has been found that taurine can stimulate the secretion of LH, which potentially induces T secretion, promotes spermatogenesis, and improves the sperm quality in testis (38).

Apart from regulating the hypothalamus and pituitary hormone release, taurine can also directly act on the testis. Abundant taurine existing in testis not only comes from endogenous synthesis in testis stated above, but also can be transported into testis across blood-testis barrier through its special transporters such as SLC6A6, also named as TauT (39).On the one hand, taurine can directly promote the secretion of T *via* acting on the Leydig cells of testis (30). In addition, an *in vitro* experiment (40) shows that taurine in interstitial cells can also increase T synthesis by enhancing autophagy process and inhibiting apoptosis. Moreover, taurine can enhance spermatogenesis. For instance, long-term oral taurine administration can increase the content and activity of lactate dehydrogenase and promote spermatogenesis of rats (41). Another study (42) has also shown that taurine-mediated Spo11a expression and meiotic initiation is necessary for germ cell mitosis. Therefore, taurine may have important applying potential in maintaining testicular physiological function.

Taurine in semen also plays an important role in keeping the physiological function of sperm. A recent metabolomic analysis in animal experiments (43) showed that extracellular low taurine concentration was associated with low fertility. *In vitro* experiments (14, 17, 44, 45) reported that taurine was necessary to maintain normal physiological functions of sperm, including sperm motility, capacitation and acrosome reaction, and the mechanism was related to its antioxidant ability. In addition, in the fallopian tube, taurine can also promote conception by inhibiting Na^+^-K^+^ ATPase activity and reducing extracellular K^+^ influx, as high K^+^ levels in the fallopian tube inhibit sperm motility and fertility (16).

## 3 The role of taurine transporter in testis and sperm

Taurine transports plasma taurine to the cell *via* taurine transporters (TauT) on the cell membrane. Taurine degradation is very slow, and it regulates the “homeostasis” of the body’s taurine mainly through intracellular synthesis and extracellular transport of taurine to exert the biological effects of taurine. Two typical taurine transporters (TauT) have been demonstrated. Solute carrier family 6 membrane 6 (SLC6A6, also named as TauT) is the most important transporter protein, with ion (sodium or chloride) dependence, high affinity and low capacity for its substrate. It is widely distributed in many organs (such as placenta and skeletal muscle (46), heart, lung, brain, liver, etc.) (47). In contrast, SLC36A1 (PAT1 transporter) is considered a proton-coupled/pH-dependent transporter, with high capacity and low affinity for the substrate, also capable of transporting other substrates (such as betaine, glycine, proline) (48). PAT1 was found to be present in different organs (e.g., heart, skeletal muscle, liver, kidney, and testis). In addition, other carriers may also be involved in the transport of taurine. For example, GABA protein can be used as taurine transporter in kidney (49), but the process and mechanism are still unclear. Taurine transporters play an important role in taurine transporting of male reproductive system. Firstly, according to the study of uptake and expression, it is found that TauT participates in the transport of taurine in the blood-testis barrier (BTB) and contributes to the internal transport of taurine in BTB to a great extent (39). Thus, TauT may play an important role in protecting germ cells from oxidative stress by transporting taurine to seminiferous tubules. Then, in the epididymis, Western blotting revealed that TauT may be involved in taurine regulation in the normal epididymis and in the proximal accumulation of taurine in the c-ros receptor tyrosine kinase-deficient sterile males (50). Additionally, previous studies have shown that taurine synthesis key enzymes are not expressed in sperm, so that sperm may rely more closely on a high-affinity TauT to obtain sufficient taurine to maintain its biological function (10, 51). It is reported that taurine and its transporter TauT are involved in the initiation of meiosis of germ cells in Japanese eel testis (42). The latest study found that TauT expression in the sperm of dyszoospermia is lower than normal (52), possibly inhibiting the uptake of cellular taurine and thus leading to abnormal increases in sperm production. Furthermore, TauT may be involved in spermatogenesis by immunofluoresogenic staining, but the specific molecular mechanisms remain to be elucidated (52). Therefore, TauT plays a key role in regulating taurine concentration, sperm quality, and spermatogenesis in the testes and in the epididymis.

## 4 The protective role of taurine in the pathological injury of the male reproductive system

### 4.1 The protective role of taurine in sperm preservation *in vitro*

#### 4.1.1 Hypothermic preservation

Semen preservation is the key to *in vitro* fertilization and artificial insemination, usually including hypothermic preservation (typically above 0°C but below normothermic 32°C to 37°C mammalian temperatures) and cryopreservation(−196 to −80 °C) (53). Hypothermic preservation, without special needs of temperature control or refrigeration equipment, has the advantages of low cost, simple operation, and is suitable for short-term preservation of various animal semen (54). Additionally, hypothermic preservation is an effective alternative method to avoid the rapid decline of sperm viability after cryopreservation (55). However, during the preservation of semen at hypothermia, the ability of the sperm’s own antioxidant defense systems is reduced and unable to balance excess ROS (54), causing oxidative stress, resulting in decreased sperm quality (loss of membrane integrity, reduced motility), loss of acrosome integrity and DNA fragmentation (56). Encouragingly, several studies have shown that taurine may be an effective additive to improve the quality of short-term semen preservation *in vitro*. For example, a previous study (57) showed that taurine (25mM) could protect ram sperm from oxidative damage when stored at a lower temperature (i.e., 4°C) for up to 72 h. Besides, the latest research (54) shows that taurine (10, 20, 40, 80, 100mM) at room temperature (i.e., 15°C) within 7 days can effectively improve the sperm quality and plasma membrane integrity of hu Sheep sperm by reducing MDA and increasing SOD, CAT, MMP activities, especially 20 mM taurine performed best. In general, the lower the preservation temperature, the better the protective effect of taurine on sperm. Moreover, at higher temperatures, taurine may not be effective in protecting sperm quality. For instance, keeping rabbit sperm at higher temperatures (i.e., 37°C), taurine does not improve the quality of sperm (e.g., motility, morphology, and acrosome integrity), even in a shorter time (i.e., 4h) (58). In addition, apart from the use of taurine alone, combinations of taurine and other elements may produce better effects. For example, according to a new study (59) discovered that the combination of taurine (25 mM) and caffeine (2 mM) at different time points (24, 48, 72 and 96 h) during short-term cold storage (i.e., 4°C) can have a significant positive effect on maintaining sperm motility, while taurine alone (25 or 50 mM) can only protect sperm motility for 24 and 48 h. In contrast, the combination prolongs the protective effect of sperm motility. The mechanism of the synergistic effect of caffeine and taurine may be that taurine protects the integrity of sperm membrane by sequestering ROS and reducing LOPs (60). Then, caffeine inhibits phosphodiesterase, making sperm cells use cAMP as energy, thereby increasing the vitality of healthier sperm cells (61).

#### 4.1.2 Cryopreservation

Sperm cryopreservation is an effective approach to long-term management and preservation of male fertility in humans and livestock (62).Cryopreservation is the process of preserving organs, tissues, cells, organelles or other biological constructs that are susceptible to damage by unregulated chemical kinetics by cooling to extremely low temperatures (generally -80°C using solid carbon dioxide or -196°C using LN_2_) (63). Especially at the temperature of -196°C, all chemical reactions, biological processes and physical intracellular and extracellular activities of liquid nitrogen are suspended. Thus, it may be an effective way to preserve sperm as long as possible. However, cryopreservation and thawing processes can damage sperm quality. Apart from freezing risks caused by osmotic stress, cold shock, intracellular ice crystal formation and dehydration, the injury in sperm cryopreservation is mostly due to excessive production of ROS (e.g., H_2_O_2_, O^2−^ and OH^−^) during sperm freeze-thaw process (64, 65) and beyond the ability of the animal’s endogenous antioxidant system to remove ROS. The imbalance between ROS production and elimination induced oxidative stress (66), resulting in loss of membrane structure and functional integrity, increased membrane permeability, apoptosis and DNA structural damage and thus decreased sperm quality including morphology, motility and viability (67–69). In order to overcome this shortcoming, researchers constantly explored adding various antioxidants to frozen supplements to reduce these damages to sperm for several decades. It had been proved that adding suitable antioxidants to semen thinners on the basis of cryopreservation media such as mainly composed of buffer (Tris), non-consisting of osmotic cryoprotectant (yolk), osmotic cryoprotectant (glycerol) and energy source (glucose) (70), can effectively reduce oxidative stress, thereby protecting sperm during freezing and thawing (64). At present, the commonly used antioxidants are divided into enzymatic antioxidants(glutathione reductase, SOD and catalase etc.)and non-enzymatic antioxidants (ascorbic acid,vitamin E, β-carotene etc.) (64). However, these antioxidants are not completely satisfactory and it is still necessary to develop newer cryoprotective additives.

A growing number of studies have shown that taurine has a good protective effect on cryopreservation of sperm quality in a variety of different species (domestic, wild or hybrid species) (**Table 1**). In contrast, the effective dose of taurine in sperm of mammals such as cattle, sheep, monkeys, and horses are generally higher than in fish (84, 85) and poultry like chickens (86). For instance, adding taurine (2 mM) to the bull sperm freezing extender did not result in an increase in semen quality metrics (76) because the taurine content was too low to reach an effective dosage. Besides, apart from a certain dose requirement, the dosage of taurine may also be affected by other factors, such as differences in diluent osmotic pressure, sperm membrane tolerance to osmotic pressure and freeze-thaw temperature etc. Furthermore, taurine protects sperm in different freezing procedures, including equiaxed freezing (71–75, 77, 79, 82, 83) and directed freezing techniques (76, 78, 81).

**Table 1:** Protective effect of taurine on cryopreservation of sperm.

Species	Treatment	Storage temperature (°C)/ duration	effects and mechanisms	Ref
Buffalo	50 mM taurine	Equilibrated at 4°C for 4 h; 5 cm above LN_2_ for 10 min; storage in LN_2_ for 6–8 weeks	Improving the motility, viability and membrane integrity of the thawed sperm, reducing the capacitation of the frozen sperm by ↑ total antioxidant status, GSH, GSH-Px, CAT and SOD	(71)
Buffalo	50 mM taurine	Equilibrated at 4°C for 4 h; 5 cm above LN_2_, for 10 min; storage in LN_2_ for 4 weeks	Increasing the sperm motility, viability and membrane integrity of post-thaw and reducing frozen capacitation of sperm by ↓intracellular Ca^2+^, cAMP and DAG	(72)
Buffalo and Cattle	50 mM taurine	Equilibrated at 4°C for 4 h; 5 cm above LN_2_ for 10 min; storage in LN_2_ for 3–4 weeks	Increasing the sperm motility, viability, membrane integrity of post-thaw; reducing the degree of frozen capacitation of sperm by ↓ protein tyrosine phosphorylation and immunolocalization	(73)
Crossbred cattle	50 mM taurine	Equilibrated at 4°C for 4 h; 5 cm above LN_2_ for 10 min; storage in LN_2_ for 3–4 weeks	Increasing the activity, viability and membrane integrity of the thawed sperm and reducing the degree of frozen capacitation of sperm by ↓H_2_O_2_, LOPs, intracellular Ca^2+^	(74)
Bubalus bubalis	50 mM taurine	Equilibrated at 4°C for 4h (cooling rate 0.3°C/min);5 cm above LN_2_, for 10 min; storage in LN_2_ for 4 weeks	Improving post-thaw motility, viability, membrane integrity of spermatozoa by ↓ tyrosine phosphorylation of sperm proteins	(75)
Bull	2 mM taurine	Cooled down to 4°C for 2h; frozen at a rate of -3°C/min from +4 to -10 C; -40°C/min from -10 to -100°C; -20°C/min from -100 to-140°C; then plunged into LN_2_ for 24h	Addition of taurine did not cause any further improvement in sperm quality	(76)
Donkey	20,40,60mM taurine	Cooling from 20 to 5°C, for 120 min 2.5cm above LN_2_ for 5 min; plunged directly into LN_2_ and storage for 1 mouth	Improving the post-thaw motility, acrosome integrity, DNA integrity of spermatozoa by↓ sperm DNA fragmentation	(77)
Crossbred Ram	40mM taurine	Equilibrated at a cold handling cabinet for 3-4 h and freezing from -5°C/min from +4 to -10 C; -40°C/min from -10 to -100 °C; -20°C/min from -100 to-140°C; then plunged into LN_2_	Improving sperm motility, live sperm percentage, integrity of sperm plasma membrane by mitigating oxidative stress: MDA↓	(78)
Ram	25,50 mM taurine	Equilibrated at 5°C for 2 h; 4.5 cm above LN_2_ for 15 min; storage in LN_2_ for 1 month	Improving the motility of thawed sperm by ↑ the activity of CAT	(79)
Ram	25,50,75,100 mM taurine	Cooled slowly to 5°C over 90-120 min frozen as 200-µL pellets on a block of solid CO_2_ and stored at −196°C in LN_2_ until required for analysis	Improving the post-thaw percentage of motile sperm through osmoregulation	(80)
Crossbred ram	40mM taurine	at 30-34°C for 3 h, to 4-5°C after 45-50 min (cooling) and freezing -5°C/min from +4 to -10 C;-40°C/min from -10 to -100 °C; -20°C/min from -100 to-140°C; then storage in LN_2_	Improving post-thaw sperm motility, live sperm count by stabilizing mitochondrial membrane and electron transport chain, (ROS, LOPs, MDA) ↓	(81)
Dog	25,50,75 mM taurine	Equilibration of 60-75 min at 4°C; 4 cm above the surface of the LN_2_ for 10 min and frozen in LN_2_ at least 1 week	Enhancing acrosome reaction by increasing sperm post-thaw motility and keeping the sperm membrane intact	(82)
Red seabrem	25,50,100 mM taurine	Equilibrated at 0°C for 5 min; frozen from 0 to −150°C at a cooling rate of 20°C/min; then transferred immediately into LN_2_	Improved the motility of frozen-thawed sperm membrane integrity, mitochondrial function by ↓ROS	(83)

In mechanisms, the protective effect of taurine on sperm in the freezing-thawing process involves multiple aspects. First, taurine can reduce sperm membrane fusion and acrosome reaction in cryopreservation through decreasing intracellular signaling molecules like Ca^2+^, cAMP and DAG for decreasing capacification-like changes in cryopreserved (72). And, taurine can also inhibit capacitation during sperm freezing by reducing sperm protein tyrosine phosphorylation levels reacting with the acrosome (75). Moreover, taurine can reduce oxidative stress by scavenging oxygen free radicals (71, 77, 78, 83), reducing the production of H_2_O_2_ (73, 74, 79) and inhibiting lipid peroxidation (73, 77, 81). Furthermore, taurine can inhibit sperm apoptosis, such as reduced DNA susceptibility to fragmentation (86). In addition, in a study on the effect of several antioxidants on the motility and fertility of ram sperm after thawing, the authors attributed the effect of taurine improving the post-thaw percentage of motile spermatozoa to osmoregulation rather than antioxidant properties (80). It was possibly because other antioxidants such as hypotaurine, carnosine or ascorbic acid could not improve the motility of thawed sperm compared with taurine.

### 4.2 The protective role of taurine on testis in ischemia reperfusion injury

Testis ischemia, commonly seen in testicular torsion, is one of the most serious urological emergencies occurring in male newborns, children, and adolescents (87). During ischemia, low-level oxygen, the decrease of cell energy storage and accumulation of toxic metabolites may lead to germ cell apoptosis (88). If testicular ischemia with severe pain is not treated within 4-6 hours, it may lead to decreased sperm motility and number, spermatogenesis disorder, infertility, testicular atrophy, and even excision (89–91). Thus, rapid diagnosis and emergency surgical detorsion are necessary to establish blood flow reperfusion of ischemic testis. Despite successful detorsion, 12%-68% of cases still suffer testicular atrophy and permanent dysfunction (92), which is due to excessive production of ROS, causing membrane lipid peroxidation, protein degeneration and DNA damage, cell dysfunction, and eventually apoptosis (93) during reperfusion. Therefore, it is equally important to minimize reperfusion injury while timely restoring blood supply to testis.

In recent years, various antioxidant substances have been studied as ROS scavengers to ameliorate the I/R injury after testicular torsion (87, 94–96). Previous studies have shown that taurine as an endogenous antidant substance has a positive effect on preventing lung (97), heart (98) and cerebral ischemia-reperfusion injury (99). Therefore, taurine is also used to prevent testicular I/R injury and significant beneficial effects have been presented in several animal experiments (**Table 2**). Firstly, taurine treatments before testicular torsion (100) or before testicular detorsion (101) can significantly reduce oxidative stress and increase spermatogenesis during testicular I/R injury. However, taurine pretreatment may have a better protective effect in histopathology (e.g., improved testicular structure and reduced desquamation and degeneration of germ cells). Compared with low doses (2h/4h, 200 mg/Kg) (101), high doses (2h/4h, 1300 mg/Kg) (102) of taurine were more effective, such as more indexes of I/R injury being improved (testicular histopathological damage and apoptosis). Further, although taurine single treatment and successive treatment can effectively improve sperm motility, sperm count and testicular antioxidant capacity, the protective effect of taurine successive treatment is obviously better than that of single dose taurine treatment (103).

**Table 2:** Protective effects of taurine on testicular I/R injury.

Species	I/R models	Treatment	Effects and mechanisms	Ref
Wistar albino rats	left testis 720°clockwise I/R(2h/2h)	250 mg/kg (Injected i.p. 1 h before detorsion)	Increasing spermatogenesis by ↓ oxidative stress, diene conjugate (DC) and protein carbonyls (PC) levels	(100)
Sprague-Dawley rats	left testis 720°counterclockwise I/R(2h/4h)	200mg/kg (Intravenously injected at repair of the testicular torsion)	Increasing spermatogenesis by ↓ myeloperoxidase activity, ROS MDA, neutrophil accumulation	(101)
Wistar rats	left testis 720°clockwise I/R(2h/4h)	1300mg/kg (Injected i.p.15 min before reperfusion)	Preventing histopathological damage by inhibiting apoptosis (TNFR 1, caspase 3 and caspase 8) ↓and ↓ NO level, eNOS expression	(102)
Sprague-Dawley rats	left testis 720°clockwise I/R(2h/7d)	300 mg/kg (Injected a single at 30min before reperfusion or continuing for 7 days)	Improving sperm viability, count by protecting antioxidant enzyme activity (SOD↑) and LOPs↓	(103)

Several mechanisms were involved in these protective effects of taurine, including reducing oxidative stress by increasing the activity of antioxidant enzymes (103) and inhibiting lipid peroxidation (100, 103) (reducing lipid peroxides diene conjugate and protein carbonyl levels) and reducing ROS production (101); anti-apoptosis by decreasing NO level and eNOS expression (102); anti-inflammatory through diminishing neutrophil recruitment to the testis (101). However, as more and more I/R models explore the mechanisms of taurine, further clinical trials are required.

### 4.3 Protective effect of taurine on diabetes-induced male reproductive dysfunction

Diabetes is one of the most common metabolic diseases, and its complications mainly include retinopathy, neuropathy, nephropathy, cardiovascular disease and decreased male fertility etc., which seriously threaten global public health (104, 105). Cumulative studies have implicated taurine in the development of diabetes mellitus and its complications (106).It has been reported that plasma taurine levels usually decrease in diabetic patients (107, 108). Further studies revealed an inverse relationship between plasma taurine levels and the parameters (such as FBG, HbA1c, and albuminuria) used for the diagnosis and follow-up of type 2 diabetes mellitus (109). Encouragingly, a growing number of studies have shown that taurine supplementation ameliorates diabetes-related complications, such as brain damage (110), neuropathy (111), retinopathy (112), etc. First, it has been demonstrated that taurine supplements have hypoglycemic, insulin sensitization and insulin secretory effects (113). The beneficial effects of taurine on type 1 diabetes have been mainly attributed to direct action on pancreatic β cells and stimulating insulin secretion. The main mechanism is that taurine can regulate the KATP channels and enhance the K-induced depolarization of pancreatic islet β cells, resulting in increased insulin secretion (114). Meanwhile, taurine also increases insulin secretion by increasing extracellular glucose concentration through the glucose transporter GLUT-2 in β cells (115). Furthermore, taurine increases Ca^2 +^ uptake of glucose by islets stimulates insulin release (116). The antidiabetic effect of taurine was also confirmed in the model of type 2 diabetes (117). Recent randomized controlled trials show that taurine can not only reduce blood glucose and blood lipids (118), but also reduce oxidative stress and inflammation in patients with type 2 diabetes (119). In addition, taurine supplementation potentially improves diabetic complications (including cardiomyopathy, nephropathy, neuropathy, retinopathy, and atherosclerosis) (120). It is well known that the occurrence and development of diabetic complications are related to oxidative stress (121). For example, diabetic nephropathy is the most common and refractory diabetic microvascular complication, and taurine can prevent kidney injury and fibrosis in diabetic animals by inhibiting glucose and AGE-induced ROS in the kidney (122). Moreover, taurine supplementation can also reduce the oxidative stress in the nerves and accelerate the speed of neurotransmission, and improve the intraneural blood circulation (123). Currently, it is well established that the protective effect of taurine treatment on male reproductive function in experimental diabetic animals. For instance, several studies (124–126) have shown that taurine reduces testicular tissue damage, DNA damage and apoptotic cells count by reducing hyperglycemia, oxidative stress (enhanced the antioxidant enzyme SOD, CAT, GPx activity) and inhibiting inflammation (reduced pro-inflammatory cytokine TNF-α and IL-6), ER stress (reduced expression of calpain-1, caspase-12 and down-regulation of CHOP, GRP78 *via* eIF2α signaling). Moreover, Taurine also restores serum GnRH, LH, FSH and T concentrations to normal levels in streptozotocin (STZ)-induced type I diabetic rats (127). According to this study, improved spermatogenesis (increased sperm number and motility, reducing sperm abnormalities) and steroidogenesis (increased mRNA expressions of testicular steroidogenesis key enzymes StAR, 3-HSD and 17β-HSD) may be the results of ameliorated HPT dysfunction after taurine treatment. Similarly, since libido is primarily regulated by androgen, taurine can increase sexual response and mating ability by enhancing the secretory function of the HPT axis (128). On the other hand, taurine also has the effect of improving erectile dysfunction. A recent study (129) showed that taurine, as an antifibrotic drug, improves erectile dysfunction in diabetic mice by reducing penile fibrosis (inhibiting expression of TGF-1), endothelial dysfunction (upregulation of the eNOS/cGMP pathway) and the production of ECM proteins. Besides, a previous *in vitro* study (130) showed that chronic taurine treatment also could prevent the development of cavernosal dysfunction after diabetes induction. In addition, for the early diabetic phase in acute high-dose STZ-induced diabetic mice, taurine pretreatment can effectively mitigate STZ-induced lipid peroxidation and ROS levels in testis and epididymal sperm (131). Even, a follow-up study suggests (132) that taurine pretreatment of male obese mice attenuated endocrine and pancreatic dysfunction in their male offspring, thereby reducing potential risk factors for metabolic disease in the next generation. Given the above findings, taurine is expected to be a potential therapeutic drug to prevent reproductive injury in diabetic men.

## 5 The protective effects of taurine against male reproductive toxicity

Male reproductive toxicity refers to the negative effects of exogenous substances on male reproductive process, including the damage to the reproductive ability of male parent and offspring (133). Male reproductive toxicity usually comes from drugs (e.g. chemotherapeutic drugs, psychotropic drugs and anti-inflammatory drugs) (134) and environmental toxins(e.g. pesticides, metals and radiation) (135) (**Table 3**). It has been reported that long-term exposure to toxic substances can lead to repeated miscarriages, stillbirths, testicular dysfunction, abnormal sperm and impaired male fertility (153). Consequently, it is significant to study how to protect the male reproductive system from toxic hazards in treatment or daily life. As a non-toxic endogenous antioxidant, taurine has become a candidate for alleviating various reproductive toxic injuries.

**Table 3:** Ameliorating effects and mechanisms of taurine against male reproductive toxicity.

Toxicity models		Species	Treatment	Effects and mechanisms	Ref
Drug	Doxorubicin	Swiss albino rats	450 mg/kg/day, orally, for 28 days	Restoring testicular weight and sperm count, T level by inhibiting oxidative stress: SDH, G6PD, Na^+^ K^+^ and Ca^2+^ ATPases↑; androgenic enzymes (3β-HSD, 17β-HSD, StAR)↑, (MDA, GSH, ROS)↓; anti-apoptosis: Bad, Fas, caspase-8, Bid proteins↓; bcl-2 abundance, mitochondrial membrane potential↑; intracellular Ca ^2+^, protein levels of calprotease, caspase-12, DNA laddering↓	(136)
	Nandrolone Decanoate	Wistar rats	100 mg/kg/day, orally for 8 weeks	Increasing testicular weight, sperm count, Viability, motility, T level by inhibiting oxidative stress: LDH-x, SOD, steroidogenic enzymes (3β-HSD, 17β-HSD), GSH↑; MDA, NO↓; anti-inflammatory: TNF-α, ICAM-1, MMP-9 gene expression↓; anti-apoptosis: cytochrome c gene expression, caspase-3 content↓; DNA damage↓	(137)
	L-NAME	Wistar rats	100 mg,200 mg/kg/day, orally for 4 weeks	Increasing sperm number, progressive motility, T level, reducing testicular and epididymal tissue lesions by inhibiting oxidative stress: antioxidant enzymes (SOD, CAT, GPx)↑,testicular function marker enzyme (ACP, ALP and LDH)↑, GSH level,↑; myeloperoxidase activity↓;H_2_O_2_, MDA, NO↓	(138)
	Chlorpromazine	Wistar rats	150 mg/kg/day, orally for 56 days	Increasing sperm count, motility, viability, volume, spermatogenesis, epididymal sperm capacitation and acrosomal reaction by inhibiting oxidative stress: testicular membrane-bound ATPase proton pump activities (Na^+^-K^+^, Ca^2+^, Mg^2+^, H^+^ ATPase)↑;dehydrogenase activities (G6PDH, LDH-X, 3β-HSD, 17β-HSD)↑	(139)
	Ornidazole	Sprague-Dawley rats	drank 2% taurine water for 20 days	Recovering sperm count, viability, motility, reducing sperm abnormality and increasing GnRH, LH and T level by inhibiting oxidative stress: mitochondrial energy metabolism,↑; epididymal epithelium structure and secretion activity (epididymal carnitine, SA, α-Glu and ACP, and mRNA expression levels of MMP7 and IDO2) ↑; (SOD, GSH, γ-GT)↑;(ROS, MDA)↓	(140)
	Cisplatin	White albino rats	50mg,150mg,250 mg/kg, oral, for 28 days	Increasing testicular weight, sperm count and T levels by inhibiting oxidative stress: GSH↑, MDA↓; anti-apoptosis: BAX↓, BCL2↓	(141)
	Methotrexate Tamoxfine	Swiss albino rats	100mg/kg, oral, for 10 days	Increasing sperm count, motility, decreasing sperm abnormalities and chromosomal aberrations in germ cells by inhibiting oxidative stress: ROS, LOPs↓; GSH↑	(142)
	5-fluorouracil	Wistar rats	50mg,100mg/kg, oral, for 4 days	Inhibiting 5-FU-induced histological abnormalities of the testis and prostate by increasing thymidylate synthetase activity	(143)
Environmental toxins	Bisphenol A	Wistar rats	100mg/kg orally, for 4 weeks	Inhibiting testicular tissue necrosis and seminiferous tubule fluctuation by inhibiting oxidative stress: (GPx, GST, CAT, SOD)↑; MDA↓	(144)
	Formaldehyde	Sprague-Dawley rats	100mg/kg/d, gavage for 30 days	Reducing testicular histopathological changes induced by formaldehyde and raising the level of LH, T by anti-apoptosis: Bax level↓	(145)
	Fluoride (NaF)	Wistar rats	100mg,200mg/kg/ day, gavage for 45 days	Restoring the T level, sperm progressive motility, sperm count, reducing abnormal sperm with morphological defects, inhibiting histopathological changes of testis and epididymis by inhibiting oxidative stress:(SOD, CAT, GPx, GST, GSH)↑; (H_2_O_2_, MDA)↓; testicular functional marker enzymes (ACP, ALP, LDH)↑; anti-inflammatory (MPO, NO, TNF-α↓); anti-apoptosis: caspase-3 activity↓	(146)
	Endosulfan	Wistar rats	100 mg/kg/day, oral gavage, for 15 days	Restoring testicular weight, increasing sperm count, motility, viability; epididymal sperm chromatin integrity and T level by inhibiting oxidative stress: testicular steroid enzymes (3β-HSD,17β-HSD)↑; dehydrogenase (G6PDH, LDH-X)↑; testicular caspases↑; SOD, CAT, GPX, GSH levels↑; anti-apoptosis: caspase-3, cytochrome c↓; mitochondrial transmembrane potential↑	(147)
	Carbon tetrachloride	Wistar rats	100 mg/kg b.w. orally, twice weekly for 4 weeks	Reducing FSH, increasing LH, T, testicular histopathological changes by inhibiting oxidative stress: LPO, MDA ↓; CAT, GSH↑;anti-inflammatory: NO↓	(148)
	Cadmium	Wistar rats	10 mg, 25 mg, 50 mg, 75 mg, 100 mg and 150 mg/kg, orally, for 5 days	Increasing T and maintaining the normal testicular structure by↑ steroidogenic enzymes, membrane stabilizing and↓ ROS (the exact mechanism needs further investigation)	(149)
	Aluminium	Swiss mice	100mg/kg, a single i.p.	Reducing germ cell degeneration, stromal cell hyperplasia lesions and improving testicular ultrastructure by inhibiting oxidative stress (specific biochemical and molecular mechanisms is unknown)	(150)
	NaAsO2	Wistar strain	100 mg/kg body weight, once daily, orally, for 5 days	Increasing testicular weight, sperm count, reducing sperm abnormalities by inhibiting oxidative stress: (SOD, CAT, GST, GR, GPx)↑;LOPs↓; anti-inflammatory: TNF-α↓; anti-apoptosis: phospho-ERK1/2↓, phospho-p38, NF-κB ↓; cytochrome C, caspase-3↓;Bcl-2↑	(151)
	Ionizing radiation	mouse spermatocytes	20, 40, 80mM taurine for 24h	Increasing GC-2 cells viability, percentage of cell cycle arrest by inhibiting oxidative stress: Nrf 2/HO-1↓; anti-apoptosis: Fas/FasL↓	(152)

### 5.1 Protective effect of taurine on drug-induced male reproductive toxicity

#### 5.1.1 Antitumor agents

In the process of cancer treatment, many antitumor drugs usually produce some adverse effects, which will affect further clinical therapies. For example, the widely used chemotherapeutic drugs doxorubicin (DOX), cisplatin (CIS), Fluorouracil (5-FU), methotrexate (MTX) and tamoxifen (TAM) have high antitumor efficacy, meanwhile with serious damage to multiple organs (e.g., cardiomyopathy, acute renal failure, acute toxic leukoencephalopathy, hepatic steatosis and testicular toxicity) (154–158). For male reproduction, these chemotherapy drugs cause damage to male reproductive function in different degrees, such as testicular toxicity (decreased testicular weight, sperm count, plasma T and testicular histopathological changes) and genetic toxicity (chromosomal aberrations). Studies (136, 141) have shown that taurine can prevent and protect testicular abnormalities (e.g., restoring testicular weight, sperm count and T level) caused by DOX and CIS through its antioxidant and anti-apoptotic properties. In addition, taurine also restores DOX-induced decrease in the activity of testicular cell membrane Na^+^-K^+^ and Ca^2+^ ATPases due to its cell membrane-stabilizing effect. Furthermore, another study (143) reported that taurine can effectively ameliorate the morphological changes of reproductive organs (e.g., spermatogenic epithelial degeneration, vacuolization of Sertoli cells and abnormal secretion of prostate) through enhancing thymidylate synthetase, decreasing 5-FU incorporation into genetic material and restoring DNA synthesis. Even, another two commonly used anti-tumor drugs, MTX and TAM, have direct genotoxic effects mainly caused by increasing chromosomal aberrations in cells (159, 160). Fortunately, taurine supplementation can not only reduce chromosome aberration of testicular cells, but also restore sperm count and motility (142). Therefore, taurine has a strong potential in alleviating antitumor drug-induced testicular function suppression and germ cell genetic material mutation.

#### 5.1.2 Hormonal medications

Nandrolone decanoate, an anabolic androgenic steroid (AAS) medication commonly used to treat anemia, cachexia, and post-menopausal osteoporosis (161). However, it is often abused by athletes to improve their physique and sporting performance (162), ignoring hazardous side effects, such as fluid retention, virilization and male reproductive dysfunction (inhibiting spermatogenesis, testicular atrophy and erectile dysfunction) (163), especially in the case of high doses or long time. Nandrolone decanoate results in male reproductive damage by promoting testicular inflammation (164), spermatogenic cells apoptotic (165) and oxidative stress (166), etc. Taurine has shown noteworthy actions to ameliorate male reproductive toxicity. It has been reported (137) that administration of taurine significantly improved testicular toxicity and DNA damage induced by Nandrolone decanoate through improving antioxidant activities like increasing LDH-x and redox markers (MDA, NO, GSH contents, and SOD) activities, reducing inflammatory indices (TNF-α, ICAM-1 and MMP-9 gene expression) and inhibiting apoptotic (decreasing cytochrome c gene expression and caspase-3 content). Thus, more clinical trials are needed to investigate the protective effects of taurine on male reproductive toxicity for Nandrolone decanoate abusers.

#### 5.1.3 Anti-inflammatory agents

Ornidazole is an antibiotic, which is mainly used to prevent and treat postoperative and reproductive tract infection (167). The most common side effects are nausea, vomiting, metallic taste, diarrhea, and long-term use can lead to severe hepatotoxicity (168). In addition, ornidazole also has rapid and reversible antifertility effects on male reproductive function by interfering with the glycolytic pathway to affect sperm production of sufficient energy (169), thereby inhibiting other sperm functions such as sperm capacitation (170) and crossing the zona pellucida (171). Therefore, the mechanism of ornidazole induced male asthenospermia may be related to the inhibition of sperm energy metabolism, which plays an important role in sperm motility and maturation. The new study (140) shows that taurine can significantly increase sperm count through enhancing sperm mitochondrial energy metabolism and stimulating the secretion of the HPT axis. Furthermore, taurine can also improve sperm viability and motility by enhancing epididymal antioxidant capacity (increasing cauda epididymal SOD, GSH and γ-GT levels, reducing ROS and MDA production) and improving secretion activity, and maintaining epididymis microenvironment homeostasis (raising concentrations of carnitine, SA, α-Glu and ACP). Thus, taurine can be a candidate drug for rescue of ornidazole-induced asthenozoospermia.

#### 5.1.4 Vasoconstrictor agents

N-nitrol-L-arginine methyl ester (L-NAME) is a vasoconstrictor, which is commonly used to induce the animal model of experimental hypertension (172), based on its inhibiting nitric oxide enzyme and consequently resulting in chronic NO depletion (173). Hypertension is a recognized risk factor for male reproductive dysfunction (174, 175), such as erectile dysfunction (176), sperm quality impairment (177), penile and testicular morphology changes (178). Since L-NAME is non-toxic, reproductive defects including decreased T levels, decreased sperm motility, antioxidant status and histological changes of internal testicular artery (179, 180) by L-NAME mainly attribute to hypertension in model animals. Previous studies have demonstrated the beneficial effects of taurine in reducing high blood pressure (181). A recent study (138)showed that taurine can effectively treat reproductive dysfunction in L-NAME-induced hypertensive rats, such as increasing testicular and epididymal sperm number, sperm progressive motility, restoring the plasma concentrations of LH, FSH and T, as well as protecting the histo-architectures of the testis and epididymis. Thus, apart from lowering blood pressure, taurine can also improve male reproductive dysfunction mediated by hypertension in L-NAME-induced hypertension animal models. The mechanisms of restoring spermatogenic function and hormone levels are related to inhibiting inflammation (decreased MPO activity) and enhancing antioxidant capacity (increased glutathione level and antioxidant enzymes activities, such as SOD, CAT; decreased the levels of H_2_O_2_ and MDA). Furthermore, further clinical trials are needed to evaluate the protective effect of taurine on reproductive dysfunction in male hypertensive patients.

#### 5.1.5 Psychotherapeutic agents

Antipsychotics are known to be harmful to male reproductive function (182). For example, chlorpromazine (CPZ) (183) is the first generation of antipsychotics, which is mainly used to treat schizophrenia and other mental diseases. Common side effects include movement problems, sleepiness, low blood pressure upon standing and even cause the potentially permanent movement disorder, neuroleptic malignant syndrome, and low white blood cell levels (184). Due to CPZ high fat soluble, can be through a blood testis barrier into the seminiferous tubule and the genital tract (139), thus long-term use of CPZ can directly lead to serious male reproductive dysfunction including reproductive hormone secretion disorder (185), inhibited spermatogenesis, capacitation and acrosome reactions (186), decreased libido and sperm quality (reduced sperm count, volume, viability, motility and morphology) (187, 188). Previous studies (139, 186, 189, 190) have shown that CPZ-induced male reproductive toxicity through excessive production of ROS/RNS decreases testicular dehydrogenase activity and flagellar motility as well as depletion of lipids on sperm cell membranes and testicular polyunsaturated fatty acids in protein causing increased sperm membrane destabilization and fluidization. Besides, CPZ can also interact with dopaminergic receptors in the anterior pituitary gland, causing neuroendocrine changes such as hyperprolactinemia, decrease in FSH, LH, T as well as steroidogenic enzymes (191, 192). Interestingly, taurine has shown some therapeutic promise in CPZ-induced male reproductive system toxicities. The latest study (139) showed that taurine treatment can ameliorate CPZ-induced inhibition of spermatogenesis (sperm count, viability, motility and morphology), sperm capacitation and acrosomal reaction through enhancing testicular dehydrogenases (3β-HSD, 17β-HSD, G6PDH, LDH-X) and electrogenic pump (Na^+^/K^+^, Ca^2+^, Mg^2+^, H^+^-ATPase) activities. Further investigations are needed to clarify the protective effects of taurine on reproductive function in male patients treated with CPZ.

### 5.2 Protective effect of taurine on environmental toxins-induced male reproductive dysfunction

Environmental toxins are ubiquitous in modern daily life. Common environmental toxins include organic chemicals (such as herbicides, pesticides), metals and ionizing radiation. Numerous studies have found that Long-term exposure to environmental toxins can cause dysfunctions such as lung diseases (193), cancer (194), hepatic damage (195), especially the male reproductive disorders (196). For example, Bisphenol A (BPA) and formaldehyde are the widespread environmental pollutants, both of which can cause male reproductive injuries including testicular histopathological changes (decreased spermatogenic cells (144) or testicular tissue necrosis and edema (145)), testicular oxidative stress damage(reducing testicular antioxidant enzyme activity (197)) and cells apoptosis(up-regulating Bax apoptotic protein expression (145)). Taurine has shown noteworthy actions to ameliorate testicular toxicity. Recent studies (144, 145) have shown that taurine was able to meliorate the testicular tissue pathologic damage induced by BPA and formaldehyde, which possibly attribute to reducing oxidative stress (increasing antioxidant enzyme activities such as GPx, GST, CAT, SOD, reducing MDA) and apoptosis (reducing Bax protein expression). Taurine also can reduce endocrine dysfunction and restore reproductive serum hormone levels (e.g., T, LH and FSH) by formaldehyde. Again, pesticides such as Sodium Fluoride (NaF), endosulfan and carbon tetrachloride, are widely used in our daily life and hazardous to various organs including testes (198). The impairments included reduced sperm quality (sperm count and sperm morphology), disrupted reproductive hormone levels, defective sperm structure and function, and testicular tissue apoptosis (199–202). It is worth noting that taurine pretreatment and treatment can effectively ameliorate these abnormalities and the mechanisms behind these effects of taurine may be attributed to reduced oxidative stress (increasing antioxidant enzyme activity and GSH levels), inhibited apoptosis (decreasing caspase-3 activity) and reduced inflammatory response (reducing the concentration of the inflammatory marker NO) (146–148).

Chronic or acute exposure to a variety of metals is also considered as an important factor in inducing male reproductive toxicity (203). For example, cadmium, arsenic(metalloid) and aluminum are three metals of concern and induce serious reproductive damage manifested by decreased T levels, testicular sperm count and sperm motility, the activity of antioxidant enzymes and glutathione in testicular tissue along with altered testis histopathology (e.g., adverse changes in Leydig cell ultrastructure). These metal-induced reproductive toxicities are mediated by multiple mechanisms, including disruption of HPT axis regulation (204), excessive NO production (205), the reduction of mitochondrial enzyme activity (206) and the induction of oxidative stress (207). Studies (149–151) have shown that taurine can protect male reproductive system from toxicity and damage caused by these metals through reducing oxidative stress (ROS scavenging, increasing the activities of the antioxidant enzymes and glutathione) and anti-apoptosis (down-regulating the activation of mitochondrial dependent signaling molecules, up-regulating the expression of Bcl-2). In addition, administration of taurine significantly improved the histopathological changes in testes induced by aluminum (150), decreased seminiferous tubule atrophy and cellular degeneration. Hence, taurine plays a beneficial role in combating metal-induced adverse effects on testis. However, current studies on taurine improving metal-induced male reproductive dysfunction mainly focus on testicular, while related studies on hypothalamic and pituitary levels are insufficient.

Another type of environmental stressor is ionizing radiation (IR). IR is widely used in areas such as medical nuclear energy and industrial manufacturing, and poses potential risks to human health. For example, acute doses can cause radiation burns and radiation sickness, while long-term low doses can lead to cancer (208, 209). The testis, one of the most radiation-sensitive organs, can suffer significant damage from even low doses of radiation, such as sperm chromosome aberrations (210), motility decreased (211), swimming behavior impaired (212) and testis weights decreased (213). Although the mechanism of IR-induced testicular toxicity is not fully understood, researchers have been actively exploring treatment methods to improve reproductive damage caused by IR. A recent *in vitro* experiment (152) found that taurine protects mouse spermatocytes (GC-2 cells) from IR-induced damage such as inhibiting the decline of GC-2 cells viability, percentage of apoptotic cells and cell cycle arres. The protective mechanisms are attributed to increasing Nrf 2 and HO-1 expression (two components in antioxidant pathway) and inhibiting Fas/FasL pathway activation in GC-2 cells. Thus, additional investigations are required to confirm the effect of taurine *in vivo*.

## 6 The mechanisms of taurine attenuating male reproductive dysfunction

**Figure 2:**
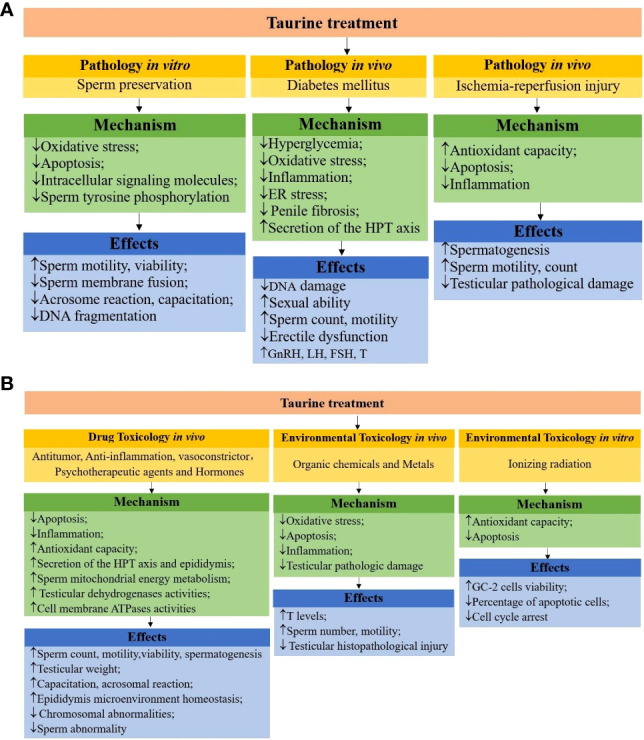
**(A)** schematic mechanisms of taurine protecting male reproductive function in pathologies. **(B)** schematic mechanisms of taurine protecting male reproductive function in toxicology.

It is known that many factors (sperm preservation *in vitro*, testicular I/R, diabetes, toxins, etc.) affect male reproductive function and many studies *in vivo* and *in vitro* have shown that taurine has a positive effect on improving male reproductive dysfunction. The relevant mechanisms are as follows (shown in **Figure 2**). First, male reproductive dysfunction is mainly caused by oxidative stress. Taurine can inhibit oxidative stress through reducing the production of oxidative substances (e.g., ROS, H_2_O_2_, MDA) or increasing the effectiveness of antioxidant defense system (increasing GSH level and the activity of antioxidant enzymes SOD, CAT, MMP, GPx). Second, taurine has anti-apoptotic (reduces eNOS, cytochrome c gene expression, caspase-3 activity, activation of the Fas/FasL pathway and phospho-ERK1/2, phospho-p38, NF-κB signaling molecules, up-regulates the expression of Bcl-2) and anti-inflammatory (reduces neutrophil recruitment, pro-inflammatory cytokine TNF- and IL-6 levels, and ICAM-1, MMP-9 gene expression) properties. Moreover, taurine can also stimulate the HPT axis secretion function and restore the normal concentrations of FSH, LH, and T. Apart from those, the protective effect of taurine involves various signaling pathways such as inhibiting ER stress (reduced expression of calpain-1, caspase-12 and down-regulation of CHOP, GRP78 *via* eIF2α signaling), decreasing intracellular signaling molecules (Ca^2+^, cAMP, DAG), increasing several cell membrane ATPases activities (e.g. Na^+^/K^+^, Ca^2+^, Mg^2+^, H^+^-ATPase). In brief, taurine can ameliorate various male reproductive dysfunction through antioxidant, anti-apoptotic, anti-inflammatory, stimulation of hormone secretion, and regulation of multiple signaling pathways.

## 7 Conclusions

As stated above, taurine is abundant in the male reproductive system, which has important physiological, pharmacological and nutritional functions to the body, and plays an important role in protecting the male reproductive system from dysfunction. In the future, it needs to be further explored and clarified whether taurine deficiency can be used as a monitoring index for male reproductive dysfunction, and whether taurine pretreatment can play a preventive role. Secondly, the current studies are mainly animal experiments. From the perspective of application, more clinical trials are needed to clarify the exact effects of taurine on human reproduction, including determining the optimal dose for maximum effect. Furthermore, there are few types of diseases related to the pathological protection of taurine, and more types of disease mechanisms need to be further explored.

## Author contributions

YL, WD, JS, and ZX collected the data and composed the manuscript. QP, SW, XG and CC helped a lot in designing the graphic abstracts. All authors have reviewed and agreed to the publication of the manuscript. All authors contributed to the article and approved the submitted version.

## References

[1] Huxtable RJ. Physiological actions of taurine. Physiol Rev (1992) 72(1):101–63. doi: 10.1152/physrev.1992.72.1.101 1731369

[2] Lambert IH Kristensen DM Holm JB Mortensen OH. Physiological role of taurine–from organism to organelle. Acta Physiol (Oxf) (2015) 213(1):191–212. doi: 10.1111/apha.12365 25142161

[3] Wojcik OP Koenig KL Zeleniuch-Jacquotte A Costa M Chen Y. The potential protective effects of taurine on coronary heart disease. Atherosclerosis (2010) 208(1):19–25. doi: 10.1016/j.atherosclerosis.2009.06.002 19592001 PMC2813349

[4] Caine JJ Geracioti TD. Taurine, energy drinks, and neuroendocrine effects. Cleve Clin J Med (2016) 83(12):895–904. doi: 10.3949/ccjm.83a.15050 27938518

[5] Froger N Moutsimilli L Cadetti L Jammoul F Wang QP Fan Y. Taurine: The comeback of a neutraceutical in the prevention of retinal degenerations. Prog Retin Eye Res (2014) 41:44–63. doi: 10.1016/j.preteyeres.2014.03.001 24721186

[6] Basili M Pedro B Hodgkiss-Geere H Navarro-Cubas X Graef N Dukes-McEwan J. Low plasma taurine levels in English cocker spaniels diagnosed with dilated cardiomyopathy. J Small Anim Pract (2021) 62(7):570–9. doi: 10.1111/jsap.13306 33594697

[7] Lake N Wright ED Lapp WS. Effects of taurine deficiency on immune function in mice. Adv Exp Med Biol (1992) 315:241–3. doi: 10.1007/978-1-4615-3436-5_28 1509945

[8] Sturman JA. Dietary taurine and feline reproduction and development. J Nutr (1991) 121(11 Suppl):S166–70. doi: 10.1093/jn/121.suppl_11.S166 1941217

[9] de la Rosa J Stipanuk MH. Evidence for a rate-limiting role of cysteinesulfinate decarboxylase activity in taurine biosynthesis in vivo. Comp Biochem Physiol B (1985) 81(3):565–71. doi: 10.1016/0305-0491(85)90367-0 4028681

[10] Li JH Ling YQ Fan JJ Zhang XP Cui S. Expression of cysteine sulfinate decarboxylase (Csd) in Male reproductive organs of mice. Histochem Cell Biol (2006) 125(6):607–13. doi: 10.1007/s00418-005-0095-8 16252094

[11] Jong CJ Ito T Mozaffari M Azuma J Schaffer S. Effect of beta-alanine treatment on mitochondrial taurine level and 5-taurinomethyluridine content. J Biomed Sci (2010) 17. doi: 10.1186/1423-0127-17-S1-S25 PMC2994391 20804600

[12] Jong CJ Sandal P Schaffer SW. The role of taurine in mitochondria health: More than just an antioxidant. Molecules (Basel, Switzerland) (2021) 26(16). doi: 10.3390/molecules26164913 PMC8400259 34443494

[13] Rezaee-Tazangi F Zeidooni L Rafiee Z Fakhredini F Kalantari H Alidadi H. Taurine effects on bisphenol a-induced oxidative stress in the mouse testicular mitochondria and sperm motility. JBRA Assist Reprod (2020) 24(4):428–35. doi: 10.5935/1518-0557.20200017 PMC7558901 32550655

[14] Meizel S Lui CW Working PK Mrsny RJ. Taurine and hypotaurine: Their effects on motility, capacitation and the acrosome reaction of hamster sperm in vitro and their presence in sperm and reproductive tract fluids of several mammals. Dev Growth Differ (1980) 22:483–94. doi: 10.1111/j.1440-169x.1980.00483.x 37281557

[15] Meizel S. Molecules that initiate or help stimulate the acrosome reaction by their interaction with the mammalian sperm surface. Am J Anat (1985) 174(3):285–302. doi: 10.1002/aja.1001740309 3934955

[16] Mrsny RJ Meizel S. Inhibition of hamster sperm na+, k+-atpase activity by taurine and hypotaurine. Life Sci (1985) 36(3):271–5. doi: 10.1016/0024-3205(85)90069-4 2981386

[17] Fraser LR. Both taurine and albumin support mouse sperm motility and fertilizing ability in vitro but there is no obligatory requirement for taurine. (Basel, Switzerland) J Reprod Fertil (1986) 77(1):271–80. doi: 10.1530/jrf.0.0770271 3755176

[18] Bosgelmez II Guvendik G. Beneficial effects of n-Acetyl-L-Cysteine or taurine pre- or post-treatments in the heart, spleen, lung, and testis of hexavalent chromium-exposed mice. Biol Trace Elem Res (2019) 190(2):437–45. doi: 10.1007/s12011-018-1571-z 30417263

[19] Casslen BG. Free amino acids in human uterine fluid. possible role of high taurine concentration. J Reprod Med (1987) 32(3):181–4. 3572897

[20] van der Horst CJ Brand A. Occurrence of hypotaurine and inositol in the reproductive tract of the ewe and its regulation by pregnenolone and progesterone. Nature (1969) 223(5201):67–8. doi: 10.1038/223067a0 5792431

[21] van der Horst CJG. Hypotaurine in the Reproductive Tract. In: Cavallini D, Gaull GE, Zappia V, editors. Natural Sulfur Compounds: Novel Biochemical and Structural Aspects. (Boston, MA: Springer US) (1980) p. 225–34. doi: 10.1007/978-1-4613-3045-5_19

[22] Buff S Donze A Guerin P Guillaud J Fontbonne A Menezo Y. Taurine and hypotaurine in spermatozoa and epididymal fluid of cats. J Reprod Fertil Suppl (2001) 57:93–5. 11787195

[23] van der Horst CJ Grooten HJ. The occurrence of hypotaurine and other sulfur-containing amino acids in seminal plasma and spermatozoa of boar, bull and dog. Biochim Biophys Acta (1966) 117(2):495–7. doi: 10.1016/0304-4165(66)90107-3 6006677

[24] Johnson LA Pursel VG Gerrits RJ Thomas CH. Free amino acid composition of porcine seminal, epididymal and seminal vesicle fluids. J Anim Sci (1972) 34(3):430–4. doi: 10.2527/jas1972.343430x 5010631

[25] Kochakian CD. Free amino acids of sex organs of the mouse: Regulation by androgen. Am J Physiol (1975) 228(4):1231–5. doi: 10.1152/ajplegacy.1975.228.4.1231 1169001

[26] Lobo MV Alonso FJ del Rio RM. Immunohistochemical localization of taurine in the Male reproductive organs of the rat. J Histochem Cytochem (2000) 48(3):313–20. doi: 10.1177/002215540004800301 10681385

[27] Holmes RP Goodman HO Shihabi ZK Jarow JP. The taurine and hypotaurine content of human semen. J Androl (1992) 13(3):289–92. doi: 10.1002/j.1939-4640.1992.tb00317.x 1601750

[28] Hernvann A Gonzales J Troupel S Galli A. Amino acid content of human semen in normal and infertility cases. Andrologia (1986) 18(5):461–9. doi: 10.1111/j.1439-0272.1986.tb01810.x 3800004

[29] Holmes RP Goodman HO Hurst CH Shihabi ZK Jarow JP. Hypotaurine in Male reproduction. Adv Exp Med Biol (1992) 315:437–41. doi: 10.1007/978-1-4615-3436-5_53 1509963

[30] Yang J Wu G Feng Y Sun C Lin S Hu J. Csd mrna expression in rat testis and the effect of taurine on testosterone secretion. Amino Acids (2010) 39(1):155–60. doi: 10.1007/s00726-009-0388-7 19921479

[31] Fan JJ Zhou JL Li JH Cui S. Accessory sex glands of Male mice have the ability to synthesize taurine *Via* the cysteine sulfinate decarboxylase pathway. Cell Biol Int (2009) 33(6):684–9. doi: 10.1016/j.cellbi.2009.03.004 19341809

[32] Park T Jerkins AA Steele RD Rogers QR Morris JG. Effect of dietary protein and taurine on enzyme activities involved in cysteine metabolism in cat tissues. J Nutr (1991) 121(11 Suppl):S181–2. doi: 10.1093/jn/121.suppl_11.S181 1941224

[33] Knopf K Sturman JA Armstrong M Hayes KC. Taurine: An essential nutrient for the cat. J Nutr (1978) 108(5):773–8. doi: 10.1093/jn/108.5.773 641594

[34] Asano A Roman HB Hirschberger LL Ushiyama A Nelson JL Hinchman MM. Cysteine dioxygenase is essential for mouse sperm osmoadaptation and Male fertility. FEBS J (2018) 285(10):1827–39. doi: 10.1111/febs.14449 PMC5992081 29604178

[35] Higuchi M Celino FT Tamai A Miura C Miura T. The synthesis and role of taurine in the Japanese eel testis. Amino Acids (2012) 43(2):773–81. doi: 10.1007/s00726-011-1128-3 22045384

[36] Kaprara A Huhtaniemi IT. The hypothalamus-Pituitary-Gonad axis: Tales of mice and men. Metabolism (2018) 86:3–17. doi: 10.1016/j.metabol.2017.11.018 29223677

[37] Feleder C Jarry H Leonhardt S Moguilevsky JA Wuttke W. Evidence to suggest that gonadotropin-releasing hormone inhibits its own secretion by affecting hypothalamic amino acid neurotransmitter release. Neuroendocrinology (1996) 64(4):298–304. doi: 10.1159/000127132 8895859

[38] Yang J Wu G Feng Y Lv Q Lin S Hu J. Effects of taurine on Male reproduction in rats of different ages. J BioMed Sci (2010) 17 Suppl 1:S9. doi: 10.1186/1423-0127-17-S1-S9 20804629 PMC2994374

[39] Kubo Y Ishizuka S Ito T Yoneyama D Akanuma SI Hosoya KI. Involvement of Taut/Slc6a6 in taurine transport at the blood-testis barrier. Metabolites (2022) 12(1). doi: 10.3390/metabo12010066 PMC8782047 35050188

[40] Yahyavy S Valizadeh A Saki G Khorsandi L. Taurine induces autophagy and inhibits oxidative stress in mice leydig cells. JBRA Assist Reprod (2020) 24(3):250–6. doi: 10.5935/1518-0557.20190079 PMC7365531 32155016

[41] Ostapiv RD Humenyuk SL Manko VV. Activity and isozyme content of lactate dehydrogenase under long-term oral taurine administration to rats. Ukr Biochem J (2015) 87(4):54–62. doi: 10.15407/ubj87.04.054 26547964

[42] Higuchi M Miura C Iwai T Miura T. Trypsin regulates meiotic initiation in the Japanese eel (Anguilla japonica) by promoting the uptake of taurine into germ cells during spermatogenesis. Biol Reprod (2013) 89(3):58. doi: 10.1095/biolreprod.113.109777 23926282

[43] DasGupta M Kumaresan A Saraf KK Paul N Sajeevkumar T Karthikkeyan G. Deciphering metabolomic alterations in seminal plasma of crossbred (Bos Taurus X bos indicus) bulls through comparative deep metabolomic analysis. Andrologia (2022) 54(1):e14253. doi: 10.1111/and.14253 34549825

[44] Mrsny RJ Waxman L Meizel S. Taurine maintains and stimulates motility of hamster sperm during capacitation in vitro. J Exp Zool (1979) 210(1):123–8. doi: 10.1002/jez.1402100113 536709

[45] Castaneda E Bouchard P Saling P Phillips D Gagnon C Bardin CW. Endogenous protein carboxyl methylation in hamster spermatozoa: Changes associated with capacitation in vitro. Int J Androl (1983) 6(5):482–96. doi: 10.1111/j.1365-2605.1983.tb00562.x 6654520

[46] Ramamoorthy S Leibach FH Mahesh VB Han H Yang-Feng T Blakely RD. Functional characterization and chromosomal localization of a cloned taurine transporter from human placenta. Biochem J (1994) 300(Pt 3):893–900. doi: 10.1042/bj3000893 8010975 PMC1138249

[47] Jhiang SM Fithian L Smanik P McGill J Tong Q Mazzaferri EL. Cloning of the human taurine transporter and characterization of taurine uptake in thyroid cells. FEBS Lett (1993) 318(2):139–44. doi: 10.1016/0014-5793(93)80008-i 8382624

[48] Thondorf I Voigt V Schäfer S Gebauer S Zebisch K Laug L. Three-dimensional quantitative structure-activity relationship analyses of substrates of the human proton-coupled amino acid transporter 1 (Hpat1). Bioorg Med Chem (2011) 19(21):6409–18. doi: 10.1016/j.bmc.2011.08.058 21955456

[49] Sivakami S Ganapathy V Leibach FH Miyamoto Y. The gamma-aminobutyric acid transporter and its interaction with taurine in the apical membrane of the bovine retinal pigment epithelium. Biochem J (1992) 283(Pt 2):391–7. doi: 10.1042/bj2830391 PMC1131046 1575683

[50] Xu YX Wagenfeld A Yeung CH Lehnert W Cooper TG. Expression and location of taurine transporters and channels in the epididymis of infertile c-ros receptor tyrosine kinase-deficient and fertile heterozygous mice. Mol Reprod Dev (2003) 64(2):144–51. doi: 10.1002/mrd.10250 12506346

[51] Huxtable RJ Lippincott SE. Diet and biosynthesis as sources of taurine in the mouse. J Nutr (1982) 112(5):1003–10. doi: 10.1093/jn/112.5.1003 7077412

[52] Wu H Zhang X Yang J Feng T Chen Y Feng R. Taurine and its transporter taut positively affect Male reproduction and early embryo development. Hum Reprod (2022) 37(6):1229–43. doi: 10.1093/humrep/deac089 PMC9156853 35526154

[53] Ma Y Gao L Tian Y Chen P Yang J Zhang L. Advanced biomaterials in cell preservation: Hypothermic preservation and cryopreservation. Acta Biomater (2021) 131:97–116. doi: 10.1016/j.actbio.2021.07.001 34242810

[54] Zhang L Wang Y Sohail T Kang Y Niu H Sun X. Effects of taurine on sperm quality during room temperature storage in hu sheep. Anim (Basel) (2021) 11(9). doi: 10.3390/ani11092725 PMC8470579 34573691

[55] Sato M Ishikawa A. Room temperature storage of mouse epididymal spermatozoa: Exploration of factors affecting sperm survival. Theriogenology (2004) 61(7-8):1455–69. doi: 10.1016/j.theriogenology.2003.07.013 15036976

[56] Al-Mutary MG Al-Ghadi MQ Ammari AA Al-Himadi AR Al-Jolimeed AH Arafah MW. Effect of different concentrations of resveratrol on the quality and in vitro fertilizing ability of ram semen stored at 5 degrees c for up to 168 h. Theriogenology (2020) 152:139–46. doi: 10.1016/j.theriogenology.2020.05.001 32408027

[57] Rather HA Islam R Malik AA Lone FA. Addition of antioxidants improves quality of ram spermatozoa during preservation at 4°C. Small Ruminant Res (2016) 141:24–8. doi: 10.1016/j.smallrumres.2016.06.007

[58] Paal D Strejcek F Tvrda E Vasicek J Balazi A Chrenek P. Taurine does not improve the quality of short-term stored rabbit spermatozoa in vitro. Reprod Domest Anim (2017) 52(6):1046–51. doi: 10.1111/rda.13022 28695635

[59] Ramirez-Perez H Guerrero-Netro HM Torres-Rodriguez P Diaz-Duran M Boeta-Acosta AM Diaw M. A combination of taurine and caffeine maintains sperm quality in equine semen during chilled storage. J Adv Vet Anim Res (2021) 8(4):635–41. doi: 10.5455/javar.2021.h555 PMC8757662 35106304

[60] Bucak MN Tekin N. Protective effect of taurine, glutathione and trehalose on the liquid storage of ram semen. Small Ruminant Res (2007) 73(1-3):103–8. doi: 10.1016/j.smallrumres.2006.12.001

[61] Špaleková E Makarevich AV Kubovičová E. Effect of caffeine on functions of cooling-stored ram sperm *in vitro* . Acta Vet Brno (2014) 83(1):19–25. doi: 10.2754/avb201483010019

[62] Hezavehei M Sharafi M Kouchesfahani HM Henkel R Agarwal A Esmaeili V. Sperm cryopreservation: A review on current molecular cryobiology and advanced approaches. Reprod BioMed Online (2018) 37(3):327–39. doi: 10.1016/j.rbmo.2018.05.012 30143329

[63] Pegg DE. Principles of cryopreservation. Methods Mol Biol (2007) 368:39–57. doi: 10.1007/978-1-59745-362-2_3 18080461

[64] Amidi F Pazhohan A Shabani Nashtaei M Khodarahmian M Nekoonam S. The role of antioxidants in sperm freezing: A review. Cell Tissue Bank (2016) 17(4):745–56. doi: 10.1007/s10561-016-9566-5 27342905

[65] Naseer Z Ahmad E Aksoy M Kucuk N Serin I Ceylan A. Protective effect of cholesterol-loaded cyclodextrin pretreatment against hydrogen peroxide induced oxidative damage in ram sperm. Cryobiology (2015) 71(1):18–23. doi: 10.1016/j.cryobiol.2015.06.007 26100676

[66] Dowling DK Simmons LW. Reactive oxygen species as universal constraints in life-history evolution. Proc Biol Sci (2009) 276(1663):1737–45. doi: 10.1098/rspb.2008.1791 PMC2674489 19324792

[67] Aitken RJ Baker MA. Oxidative stress and Male reproductive biology. Reprod Fertil Dev (2004) 16(5):581–8. doi: 10.10371/RD03089 15367373

[68] Gadea J Selles E Marco MA Coy P Matas C Romar R. Decrease in glutathione content in boar sperm after cryopreservation. effect of the addition of reduced glutathione to the freezing and thawing extenders. Theriogenology (2004) 62(3-4):690–701. doi: 10.1016/j.theriogenology.2003.11.013 15226023

[69] Hsieh YY Chang CC Lin CS. Seminal malondialdehyde concentration but not glutathione peroxidase activity is negatively correlated with seminal concentration and motility. Int J Biol Sci (2006) 2(1):23–9. doi: 10.7150/ijbs.2.23 PMC1457038 16680200

[70] AbdelHafez F Bedaiwy M El-Nashar SA Sabanegh E Desai N. Techniques for cryopreservation of individual or small numbers of human spermatozoa: A systematic review. Hum Reprod Update (2009) 15(2):153–64. doi: 10.1093/humupd/dmn061 19109313

[71] Shiva Shankar Reddy N Jagan Mohanarao G Atreja SK. Effects of adding taurine and trehalose to a tris-based egg yolk extender on buffalo (Bubalus bubalis) sperm quality following cryopreservation. Anim Reprod Sci (2010) 119(3-4):183–90. doi: 10.1016/j.anireprosci.2010.01.012 20197223

[72] Singh VK Atreja SK Kumar R Chhillar S Singh AK. Assessment of intracellular Ca2+, camp and 1,2-diacylglycerol in cryopreserved buffalo (Bubalus bubalis) spermatozoa on supplementation of taurine and trehalose in the extender. Reprod Domest Anim (2012) 47(4):584–90. doi: 10.1111/j.1439-0531.2011.01922.x 21988572

[73] Kumar R Singh VK Chhillar S Atreja SK. Effect of supplementation of taurine or trehalose in extender on immunolocalization of tyrosine phosphoproteins in buffalo and cattle (Karan fries) cryopreserved spermatozoa. Reprod Domest Anim (2013) 48(3):407–15. doi: 10.1111/rda.12088 23106450

[74] Chhillar S Singh VK Kumar R Atreja SK. Effects of taurine or trehalose supplementation on functional competence of cryopreserved karan fries semen. Anim Reprod Sci (2012) 135(1-4):1–7. doi: 10.1016/j.anireprosci.2012.08.029 22974707

[75] Kumar R Atreja SK. Effect of incorporation of additives in tris-based egg yolk extender on buffalo (Bubalus bubalis) sperm tyrosine phosphorylation during cryopreservation. Reprod Domest Anim (2012) 47(3):485–90. doi: 10.1111/j.1439-0531.2011.01908.x 22364363

[76] Sariozkan S Bucak MN Tuncer PB Ulutas PA Bilgen A. The influence of cysteine and taurine on microscopic-oxidative stress parameters and fertilizing ability of bull semen following cryopreservation. Cryobiology (2009) 58(2):134–8. doi: 10.1016/j.cryobiol.2008.11.006 19070613

[77] Bottrel M Acha D Ortiz I Hidalgo M Gosalvez J Camisao J. Cryoprotective effect of glutamine, taurine, and proline on post-thaw semen quality and DNA integrity of donkey spermatozoa. Anim Reprod Sci (2018) 189:128–35. doi: 10.1016/j.anireprosci.2017.12.021 29325880

[78] Lone FA Naikoo M Shah SM Darzi SA Farooq J. Effect of idebenone, resveratrol and taurine on the sperm quality and lipid peroxidation of cryopreserved crossbred ram semen. Cryo Lett (2021) 42(3):146–53. 33970992

[79] Bucak MN Atessahin A Varisli O Yuce A Tekin N Akcay A. The influence of trehalose, taurine, cysteamine and hyaluronan on ram semen microscopic and oxidative stress parameters after freeze-thawing process. Theriogenology (2007) 67(5):1060–7. doi: 10.1016/j.theriogenology.2006.12.004 17280711

[80] Sanchez-Partida LG Setchell BP Maxwell WM. Epididymal compounds and antioxidants in diluents for the frozen storage of ram spermatozoa. Reprod Fertil Dev (1997) 9(7):689–96. doi: 10.1071/r97045 9623488

[81] Banday MN Lone FA Rasool F Rashid M Shikari A. Use of antioxidants reduce lipid peroxidation and improve quality of crossbred ram sperm during its cryopreservation. Cryobiology (2017) 74:25–30. doi: 10.1016/j.cryobiol.2016.12.008 28040489

[82] Martins-Bessa A Rocha A Mayenco-Aguirre A. Effects of taurine and hypotaurine supplementation and ionophore concentrations on post-thaw acrosome reaction of dog spermatozoa. Theriogenology (2009) 71(2):248–53. doi: 10.1016/j.theriogenology.2008.07.006 18774600

[83] Liu Q Wang X Wang W Zhang X Xu S Ma D. Effect of the addition of six antioxidants on sperm motility, membrane integrity and mitochondrial function in red seabream (Pagrus major) sperm cryopreservation. Fish Physiol Biochem (2015) 41(2):413–22. doi: 10.1007/s10695-014-9993-9 25255938

[84] Martinez-Paramo S Diogo P Dinis MT Soares F Sarasquete C Cabrita E. Effect of two sulfur-containing amino acids, taurine and hypotaurine in European Sea bass (Dicentrarchus labrax) sperm cryopreservation. Cryobiology (2013) 66(3):333–8. doi: 10.1016/j.cryobiol.2013.04.001 23583301

[85] Kutluyer F Ogretmen F Inanan BE. Cryopreservation of goldfish (Carassius auratus) spermatozoa: Effects of extender supplemented with taurine on sperm motility and DNA damage. Cryo Lett (2016) 37(1):41–6. 26964024

[86] Partyka A Rodak O Bajzert J Kochan J Nizanski W. The effect of l-carnitine, hypotaurine, and taurine supplementation on the quality of cryopreserved chicken semen. BioMed Res Int (2017) 2017:7279341. doi: 10.1155/2017/7279341 28523277 PMC5421088

[87] Wei SM Yan ZZ Zhou J. Protective effect of rutin on testicular ischemia-reperfusion injury. J Pediatr Surg (2011) 46(7):1419–24. doi: 10.1016/j.jpedsurg.2010.09.044 21763845

[88] Kostakis ID Zavras N Damaskos C Sakellariou S Korkolopoulou P Misiakos EP. Erythropoietin and sildenafil protect against Ischemia/Reperfusion injury following testicular torsion in adult rats. Exp Ther Med (2017) 13(6):3341–7. doi: 10.3892/etm.2017.4441 PMC5450555 28587411

[89] Jacobsen FM Rudlang TM Fode M Ostergren PB Sonksen J Ohl DA. The impact of testicular torsion on testicular function. World J Mens Health (2020) 38(3):298–307. doi: 10.5534/wjmh.190037 31081295 PMC7308234

[90] Turner TT Brown KJ. Spermatic cord torsion: Loss of spermatogenesis despite return of blood flow. Biol Reprod (1993) 49(2):401–7. doi: 10.1095/biolreprod49.2.401 8373967

[91] Filho DW Torres MA Bordin AL Crezcynski-Pasa TB Boveris A. Spermatic cord torsion, reactive oxygen and nitrogen species and ischemia-reperfusion injury. Mol Aspects Med (2004) 25(1-2):199–210. doi: 10.1016/j.mam.2004.02.020 15051328

[92] Wei SM Wang RY Chen YS. Sesamol protects testis from ischemia-reperfusion injury through scavenging reactive oxygen species and upregulating cremtau expression. Oxid Med Cell Longev (2020) 2020:9043806. doi: 10.1155/2020/9043806 32655774 PMC7320277

[93] Ghasemnezhad R Mohammadghasemi F Faghani M Bahadori MH. Oxytocin can decrease germ cells apoptotic index in testis under acute ischemia reperfusion in a rat model. Iran J Reprod Med (2015) 13(5):283–90. PMC4515235 26221127

[94] Cay A Alver A Kucuk M Isik O Eminagaoglu MS Karahan SC. The effects of n-acetylcysteine on antioxidant enzyme activities in experimental testicular torsion. J Surg Res (2006) 131(2):199–203. doi: 10.1016/j.jss.2005.11.572 16412470

[95] Aktoz T Kanter M Aktas C. Protective effects of quercetin on testicular Torsion/Detorsion-induced ischaemia-reperfusion injury in rats. Andrologia (2010) 42(6):376–83. doi: 10.1111/j.1439-0272.2010.01044.x 21105888

[96] Taskara E Gor A Kutlu O Karaguzel E Cobanoglu U Topbas M. Does propofol prevent testicular ischemia-reperfusion injury due to torsion in the long term? Pediatr Surg Int (2011) 27(9):1003–7. doi: 10.1007/s00383-011-2895-3 21626015

[97] Guler L Tavlasoglu M Yucel O Guler A Sahin MA Kurkluoglu M. Taurine attenuates lung ischemia-reperfusion injury after lung transplantation in rats. J Anesth (2014) 28(3):347–53. doi: 10.1007/s00540-013-1741-2 24197293

[98] Su Q Liu Y Lv XW Ye ZL Sun YH Kong BH. Inhibition of lncrna Tug1 upregulates mir-142-3p to ameliorate myocardial injury during ischemia and reperfusion *Via* targeting Hmgb1- and Rac1-induced autophagy. J Mol Cell Cardiol (2019) 133:12–25. doi: 10.1016/j.yjmcc.2019.05.021 31145943

[99] Rukan TA Mksimovich NE Zimatkin SM. [Morphofunctional state of vessel endothelium at the early stage of cerebral ischemia-reperfusion and the effect of taurin administration]. Eksperimental’naia i klinicheskaia farmakologiia (2013) 76(12):8–10. 24605420

[100] Abbasoglu L Kalaz EB Soluk-Tekkesin M Olgac V Dogru-Abbasoglu S Uysal M. Beneficial effects of taurine and carnosine in experimental Ischemia/Reperfusion injury in testis. Pediatr Surg Int (2012) 28(11):1125–31. doi: 10.1007/s00383-012-3168-5 22961384

[101] Wei SM Yan ZZ Zhou J. Beneficial effect of taurine on testicular ischemia-reperfusion injury in rats. Urology (2007) 70(6):1237–42. doi: 10.1016/j.urology.2007.09.030 18158068

[102] Aydos TR Basar MM Kul O Atmaca HT Uzunalioglu T Kisa U. Effects of ozone therapy and taurine on Ischemia/Reperfusion-induced testicular injury in a rat testicular torsion model. Turk J Med Sci (2014) 44(5):749–55. doi: 10.3906/sag-1308-20 25539540

[103] Zhang JH Zhang YB Shi YB Lei-Ming GE Zhong-Jiao XU Zhang JP. The protective effect of taurine on the testis with ischemia reperfusion injury in prepubertal rats. Acta Nutrimenta Sin (2015) 37(03):270–4. doi: 10.13325/j.cnki.acta.nutr.sin.2015.03.016

[104] Melendez-Ramirez LY Richards RJ Cefalu WT. Complications of type 1 diabetes. Endocrinol Metab Clin North Am (2010) 39(3):625–40. doi: 10.1016/j.ecl.2010.05.009 20723824

[105] Epstein FH Atkinson MA Maclaren NK. The pathogenesis of insulin-dependent diabetes mellitus. New Engl J Med (1994) 331(21):1428–36. doi: 10.1056/NEJM199411243312107 7969282

[106] Hansen SH. The role of taurine in diabetes and the development of diabetic complications. Diabetes Metab Res Rev (2001) 17(5):330–46. doi: 10.1002/dmrr.229 11747139

[107] Franconi F Bennardini F Mattana A Miceli M Ciuti M Mian M. Plasma and platelet taurine are reduced in subjects with insulin-dependent diabetes mellitus: Effects of taurine supplementation. Am J Clin Nutr (1995) 61(5):1115–9. doi: 10.1093/ajcn/61.4.1115 7733037

[108] De Luca G Calpona PR Caponetti A Romano G Di Benedetto A Cucinotta D. Taurine and osmoregulation: Platelet taurine content, uptake, and release in type 2 diabetic patients. Metabolism (2001) 50(1):60–4. doi: 10.1053/meta.2001.19432 11172476

[109] Sak D Erdenen F Muderrisoglu C Altunoglu E Sozer V Gungel H. The relationship between plasma taurine levels and diabetic complications in patients with type 2 diabetes mellitus. Biomolecules (2019) 9(3). doi: 10.3390/biom9030096 PMC6468751 30862074

[110] Mohamed NA Gawad H. Taurine dietary supplementation attenuates brain, thyroid, testicular disturbances and oxidative stress in streptozotocin-induced diabetes mellitus in Male rats. Beni-Suef Univ J Basic Appl Sci (2017) 6(3):247–52. doi: 10.1016/j.bjbas.2017.04.006

[111] Agca CA Tuzcu M Hayirli A Sahin K. Taurine ameliorates neuropathy *Via* regulating nf-Kb and Nrf2/Ho-1 signaling cascades in diabetic rats. Food Chem Toxicol (2014) 71:116–21. doi: 10.1016/j.fct.2014.05.023 24907624

[112] Yu X Xu Z Mi M Xu H Zhu J Wei N. Dietary taurine supplementation ameliorates diabetic retinopathy *Via* anti-excitotoxicity of glutamate in streptozotocin-induced sprague-dawley rats. Neurochem Res (2008) 33(3):500–7. doi: 10.1007/s11064-007-9465-z 17762918

[113] Inam UL Piao F Aadil RM Suleman R Li K Zhang M. Ameliorative effects of taurine against diabetes: A review. Amino Acids (2018) 50(5):487–502. doi: 10.1007/s00726-018-2544-4 29492671

[114] Park EJ Bae JH Kim SY Lim JG Baek WK Kwon TK. Inhibition of atp-sensitive k+ channels by taurine through a benzamido-binding site on sulfonylurea receptor 1. Biochem Pharmacol (2004) 67(6):1089–96. doi: 10.1016/j.bcp.2003.11.003 15006545

[115] Carneiro EM Latorraca MQ Araujo E Beltra M Oliveras MJ Navarro M. Taurine supplementation modulates glucose homeostasis and islet function. J Nutr Biochem (2009) 20(7):503–11. doi: 10.1016/j.jnutbio.2008.05.008 18708284

[116] Ribeiro RA Vanzela EC Oliveira CA Bonfleur ML Boschero AC Carneiro EM. Taurine supplementation: Involvement of Cholinergic/Phospholipase c and protein kinase a pathways in potentiation of insulin secretion and Ca2+ handling in mouse pancreatic islets. Br J Nutr (2010) 104(8):1148–55. doi: 10.1017/S0007114510001820 20591207

[117] Nandhini AT Thirunavukkarasu V Anuradha CV. Taurine modifies insulin signaling enzymes in the fructose-fed insulin resistant rats. Diabetes Metab (2005) 31(4 Pt 1):337–44. doi: 10.1016/s1262-3636(07)70202-1 16369195

[118] Maleki V Alizadeh M Esmaeili F Mahdavi R. The effects of taurine supplementation on glycemic control and serum lipid profile in patients with type 2 diabetes: A randomized, double-blind, placebo-controlled trial. Amino Acids (2020) 52(6-7):905–14. doi: 10.1007/s00726-020-02859-8 32472292

[119] Maleki V Mahdavi R Hajizadeh-Sharafabad F Alizadeh M. The effects of taurine supplementation on oxidative stress indices and inflammation biomarkers in patients with type 2 diabetes: A randomized, double-blind, placebo-controlled trial. Diabetol Metab Syndr (2020) 12:9. doi: 10.1186/s13098-020-0518-7 32015761 PMC6990511

[120] Ito T Schaffer SW Azuma J. The potential usefulness of taurine on diabetes mellitus and its complications. Amino Acids (2012) 42(5):1529–39. doi: 10.1007/s00726-011-0883-5 PMC3325402 21437784

[121] Giacco F Brownlee M. Oxidative stress and diabetic complications. Circ Res (2010) 107(9):1058–70. doi: 10.1161/CIRCRESAHA.110.223545 PMC2996922 21030723

[122] Yao HT Lin P Chang YW Chen CT Chiang MT Chang L. Effect of taurine supplementation on cytochrome P450 2e1 and oxidative stress in the liver and kidneys of rats with streptozotocin-induced diabetes. Food Chem Toxicol (2009) 47(7):1703–9. doi: 10.1016/j.fct.2009.04.030 19406192

[123] Askwith T Zeng W Eggo MC Stevens MJ. Taurine reduces nitrosative stress and nitric oxide synthase expression in high glucose-exposed human schwann cells. Exp Neurol (2012) 233(1):154–62. doi: 10.1016/j.expneurol.2011.09.010 PMC3268940 21952043

[124] Ghosh S Chowdhury S Das AK Sil PC. Taurine ameliorates oxidative stress induced inflammation and er stress mediated testicular damage in stz-induced diabetic wistar rats. Food Chem Toxicol (2019) 124:64–80. doi: 10.1016/j.fct.2018.11.055 30496779

[125] Abd El-Twab SM Mohamed HM Mahmoud AM. Taurine and pioglitazone attenuate diabetes-induced testicular damage by abrogation of oxidative stress and up-regulation of the pituitary-gonadal axis. Can J Physiol Pharmacol (2016) 94(6):651–61. doi: 10.1139/cjpp-2015-0503 27089006

[126] Tsounapi P Saito M Dimitriadis F Koukos S Shimizu S Satoh K. Antioxidant treatment with edaravone or taurine ameliorates diabetes-induced testicular dysfunction in the rat. Mol Cell Biochem (2012) 369(1-2):195–204. doi: 10.1007/s11010-012-1382-z 22763673

[127] Liu H Lin S Lv Q Yang Q Wu G Hu J. Taurine recovers testicular steroidogenesis and spermatogenesis in streptozotocin-induced diabetic rats. Adv Exp Med Biol (2017) 975 Pt 2:801–11. doi: 10.1007/978-94-024-1079-2_62 28849500

[128] Yang J Lin S Zhang Y Wu G Yang Q Lv Q. Taurine improves sexual function in streptozotocin-induced diabetic rats. Adv Exp Med Biol (2017) 975 Pt 1:307–18. doi: 10.1007/978-94-024-1079-2_27 28849465

[129] Ruan Y Li M Wang T Yang J Rao K Wang S. Taurine supplementation improves erectile function in rats with streptozotocin-induced type 1 diabetes *Via* amelioration of penile fibrosis and endothelial dysfunction. J Sex Med (2016) 13(5):778–85. doi: 10.1016/j.jsxm.2016.02.164 27017070

[130] Dalaklioglu S Kuscu N Celik-Ozenci C Bayram Z Nacitarhan C Ozdem SS. Chronic treatment with taurine ameliorates diabetes-induced dysfunction of nitric oxide-mediated neurogenic and endothelium-dependent corpus cavernosum relaxation in rats. Fundam Clin Pharmacol (2014) 28(4):394–404. doi: 10.1111/fcp.12041 23848484

[131] Shrilatha B Muralidhara. Early oxidative stress in testis and epididymal sperm in streptozotocin-induced diabetic mice: Its progression and genotoxic consequences. . Reprod Toxicol (2007) 23(4):578–87. doi: 10.1016/j.reprotox.2007.02.001 17360155

[132] Freitas IN Dos Reis Araujo T Vettorazzi JF Magalhaes EA Carneiro EM Bonfleur ML. Taurine supplementation in high-fat diet fed Male mice attenuates endocrine pancreatic dysfunction in their Male offspring. Amino Acids (2019) 51(4):727–38. doi: 10.1007/s00726-019-02712-7 30830312

[133] Han X Huang Q. Environmental pollutants exposure and Male reproductive toxicity: The role of epigenetic modifications. Toxicology (2021) 456:152780. doi: 10.1016/j.tox.2021.152780 33862174

[134] Nudell DM Monoski MM Lipshultz LI. Common medications and drugs: How they affect Male fertility. Urol Clin N Am (2002) 29(4):965–73. doi: 10.1016/S0094-0143(02)00079-4 12516765

[135] Krzastek SC Farhi J Gray M Smith RP. Impact of environmental toxin exposure on Male fertility potential. Transl Androl Urol (2020) 9(6):2797–813. doi: 10.21037/tau-20-685 PMC7807371 33457251

[136] Das J Ghosh J Manna P Sil PC. Taurine protects rat testes against doxorubicin-induced oxidative stress as well as P53, fas and caspase 12-mediated apoptosis. Amino Acids (2012) 42(5):1839–55. doi: 10.1007/s00726-011-0904-4 21476075

[137] Ahmed MA. Amelioration of nandrolone decanoate-induced testicular and sperm toxicity in rats by taurine: Effects on steroidogenesis, redox and inflammatory cascades, and intrinsic apoptotic pathway. Toxicol Appl Pharmacol (2015) 282(3):285–96. doi: 10.1016/j.taap.2014.12.007 25542992

[138] Adedara IA Alake SE Adeyemo MO Olajide LO Ajibade TO Farombi EO. Taurine enhances spermatogenic function and antioxidant defense mechanisms in testes and epididymis of l-Name-Induced hypertensive rats. BioMed Pharmacother (2018) 97:181–9. doi: 10.1016/j.biopha.2017.10.095 29091864

[139] Oyovwi MO Nwangwa EK Ben-Azu B Rotue RA Edesiri TP Emojevwe V. Prevention and reversal of chlorpromazine induced testicular dysfunction in rats by synergistic testicle-active flavonoids, taurine and coenzyme-10. Reprod Toxicol (2021) 101:50–62. doi: 10.1016/j.reprotox.2021.01.013 33548410

[140] Du Y Liu H Zhang M Zhang S Hu J Wu G. Taurine increases spermatozoa quality and function in asthenospermia rats impaired by ornidazole. Adv Exp Med Biol (2019) 1155:507–20. doi: 10.1007/978-981-13-8023-5_47 31468427

[141] Azab SS Kamel I Ismail NN El Din Hosni H El Fatah MA. The defensive role of taurine against gonadotoxicity and testicular apoptosis effects induced by cisplatin in rats. J Infect Chemother (2020) 26(1):51–7. doi: 10.1016/j.jiac.2019.07.004 31353201

[142] Alam SS Hafiz NA Abd El-Rahim AH. Protective role of taurine against genotoxic damage in mice treated with methotrexate and tamoxfine. Environ Toxicol Pharmacol (2011) 31(1):143–52. doi: 10.1016/j.etap.2010.10.001 21787679

[143] Al-Asmari AK Al-Zahrani AM Khan AQ Al-Shahrani HM Ali Al Amri M. Taurine ameliorates 5-Flourouracil-Induced intestinal mucositis, hepatorenal and reproductive organ damage in wistar rats: A biochemical and histological study. Hum Exp Toxicol (2016) 35(1):10–20. doi: 10.1177/0960327115573597 25724421

[144] Kalender S Apaydin FG Kalender Y. Testicular toxicity of orally administrated bisphenol a in rats and protective role of taurine and curcumin. Pak J Pharm Sci (2019) 32(3):1043–7. 31278718

[145] Geng T Sun F Tian Y Xiao YH Sun C Liu B. Protective effect of taurine against formaldehyde-induced Male reproductive toxicity in adult Male rats. Zhonghua Nan Ke Xue (2020) 26(9):777–82. doi: 10.13263/j.cnki.nja.2020.09.002 33377698

[146] Adedara IA Olabiyi BF Ojuade TD Idris UF Onibiyo EM Farombi EO. Taurine reverses sodium fluoride-mediated increase in inflammation, caspase-3 activity, and oxidative damage along the brain-Pituitary-Gonadal axis in Male rats. Can J Physiol Pharmacol (2017) 95(9):1019–29. doi: 10.1139/cjpp-2016-0641 28654759

[147] Aly HA Khafagy RM. Taurine reverses endosulfan-induced oxidative stress and apoptosis in adult rat testis. Food Chem Toxicol (2014) 64:1–9. doi: 10.1016/j.fct.2013.11.007 24262488

[148] Abd-Elhakim YM Ghoneim MH Ebraheim LLM Imam TS. Taurine and hesperidin rescues carbon tetrachloride-triggered testicular and kidney damage in rats *Via* modulating oxidative stress and inflammation. Life Sci (2020) 254:117782. doi: 10.1016/j.lfs.2020.117782 32407847

[149] Manna P Sinha M Sil PC. Cadmium induced testicular pathophysiology: Prophylactic role of taurine. Reprod Toxicol (2008) 26(3-4):282–91. doi: 10.1016/j.reprotox.2008.09.009 18926901

[150] Abdel-Moneim AM. Effects of taurine against histomorphological and ultrastructural changes in the testes of mice exposed to aluminium chloride. Arh Hig Rada Toksikol (2013) 64(3):405–14. doi: 10.2478/10004-1254-64-2013-2322 24084349

[151] Das J Ghosh J Manna P Sinha M Sil PC. Taurine protects rat testes against Naaso(2)-induced oxidative stress and apoptosis *Via* mitochondrial dependent and independent pathways. Toxicol Lett (2009) 187(3):201–10. doi: 10.1016/j.toxlet.2009.03.001 19429265

[152] Yang W Huang J Xiao B Liu Y Zhu Y Wang F. Taurine protects mouse spermatocytes from ionizing radiation-induced damage through activation of Nrf2/Ho-1 signaling. Cell Physiol Biochem (2017) 44(4):1629–39. doi: 10.1159/000485762 29216642

[153] Kumar S. Occupational and environmental exposure to lead and reproductive health impairment: An overview. Indian J Occup Envir (2018) 22(3):128–37. doi: 10.4103/ijoem.IJOEM_126_18 PMC6309352 30647514

[154] Chatterjee K Zhang J Honbo N Karliner JS. Doxorubicin cardiomyopathy. Cardiology (2010) 115(2):155–62. doi: 10.1159/000265166 PMC2848530 20016174

[155] Safirstein R Winston J Goldstein M Moel D Dikman S Guttenplan J. Cisplatin nephrotoxicity. Am J Kidney Dis (1986) 8(5):356–67. doi: 10.1016/s0272-6386(86)80111-1 3538859

[156] Salkade PR Lim TA. Methotrexate-induced acute toxic leukoencephalopathy. J Cancer Res Ther (2012) 8(2):292–6. doi: 10.4103/0973-1482.98993 22842379

[157] Ribeiro MP Santos AE Custodio JB. Mitochondria: The gateway for tamoxifen-induced liver injury. Toxicology (2014) 323:10–8. doi: 10.1016/j.tox.2014.05.009 24881593

[158] Lee S Lee MS Park J Zhang JY Jin DI. Oxidative stress in the testis induced by tamoxifen and its effects on early embryo development in isogenic mice. J Toxicol Sci (2012) 37(4):675–9. doi: 10.2131/jts.37.675 22863848

[159] Padmanabhan S Tripathi DN Vikram A Ramarao P Jena GB. Methotrexate-induced cytotoxicity and genotoxicity in germ cells of mice: Intervention of folic and folinic acid. Mutat Res (2009) 673(1):43–52. doi: 10.1016/j.mrgentox.2008.11.011 19110071

[160] Vijayalaxmi KK Rai SP. Studies on the genotoxicity of tamoxifen citrate in mouse bone marrow cells. Mutat Res (1996) 368(2):109–14. doi: 10.1016/0165-1218(95)00101-8 8684400

[161] Evans NA. Current concepts in anabolic-androgenic steroids. Am J Sports Med (2004) 32(2):534–42. doi: 10.1177/0363546503262202 14977687

[162] Patane FG Liberto A Maria Maglitto AN Malandrino P Esposito M Amico F. Nandrolone decanoate: Use, abuse and side effects. Medicina (Kaunas) (2020) 56(11). doi: 10.3390/medicina56110606 PMC7696474 33187340

[163] Shokri S Aitken RJ Abdolvahhabi M Abolhasani F Ghasemi FM Kashani I. Exercise and supraphysiological dose of nandrolone decanoate increase apoptosis in spermatogenic cells. Basic Clin Pharmacol Toxicol (2010) 106(4):324–30. doi: 10.1111/j.1742-7843.2009.00495.x 20002066

[164] Hughes TK Fulep E Juelich T Smith EM Stanton GJ. Modulation of immune responses by anabolic androgenic steroids. Int J Immunopharmacol (1995) 17(11):857–63. doi: 10.1016/0192-0561(95)00078-x 8788115

[165] Mohamed HM Mohamed MA. Effect of different doses of nandrolone decanoate on lipid peroxidation, DNA fragmentation, sperm abnormality and histopathology of testes of Male wister rats. Exp Toxicol Pathol (2015) 67(1):1–11. doi: 10.1016/j.etp.2014.09.003 25440442

[166] Tahtamouni LH Mustafa NH Hassan IM Ahmad IM Abdalla MY. Nandrolone decanoate administration to Male rats induces oxidative stress, seminiferous tubules abnormalities, and sperm DNA fragmentation. Jordan J Biol Sci (2011) 3:165–74.

[167] Kuhlmann FM Fleckenstein JM. Antiparasitic agents. In: Cohen J Powderly WG Opal SM, editors. Infectious diseases. Elsevier (2017). p. 1345–72.e2.

[168] Coskun Y Erarslan E Dogan M Koc H Yigit SN Yuksel I. Severe hepatotoxicity as a result of extended use of ornidazole. J Clin Gastroenterol (2012) 46(6):529–30. doi: 10.1097/MCG.0b013e318250056d 22691836

[169] Oberlander G Yeung CH Cooper TG. Influence of oral administration of ornidazole on capacitation and the activity of some glycolytic enzymes of rat spermatozoa. J Reprod Fertil (1996) 106(2):231–9. doi: 10.1530/jrf.0.1060231 8699406

[170] Siva AB Yeung CH Cooper TG Shivaji S. Antimicrobial drug ornidazole inhibits hamster sperm capacitation, in vitro. Reprod Toxicol (2006) 22(4):702–9. doi: 10.1016/j.reprotox.2006.04.013 16777375

[171] Bone W Jones NG Kamp G Yeung CH Cooper TG. Effect of ornidazole on fertility of Male rats: Inhibition of a glycolysis-related motility pattern and zona binding required for fertilization in vitro. J Reprod Fertil (2000) 118(1):127–35. doi: 10.1530/jrf.0.1180127 10793634

[172] Saravanakumar M Raja B. Veratric acid, a phenolic acid attenuates blood pressure and oxidative stress in l-name induced hypertensive rats. Eur J Pharmacol (2011) 671(1-3):87–94. doi: 10.1016/j.ejphar.2011.08.052 21937012

[173] Bansinath M Arbabha B Turndorf H Garg UC. Chronic administration of a nitric oxide synthase inhibitor, n omega-Nitro-L-Arginine, and drug-induced increase in cerebellar cyclic gmp in vivo. Neurochem Res (1993) 18(10):1063–6. doi: 10.1007/BF00966685 7504789

[174] Nilsson PM Viigimaa M Giwercman A Cifkova R. Hypertension and reproduction. Curr Hypertens Rep (2020) 22(4):29. doi: 10.1007/s11906-020-01036-2 32170412 PMC7069900

[175] Navaneethabalakrishnan S Goodlett BL Lopez AH Rutkowski JM Mitchell BM. Hypertension and reproductive dysfunction: A possible role of inflammation and inflammation-associated lymphangiogenesis in gonads. Clin Sci (Lond) (2020) 134(24):3237–57. doi: 10.1042/CS20201023 PMC9208534 33346358

[176] Giuliano FA Leriche A Jaudinot EO de Gendre AS. Prevalence of erectile dysfunction among 7689 patients with diabetes or hypertension, or both. Urology (2004) 64(6):1196–201. doi: 10.1016/j.urology.2004.08.059 15596196

[177] Colli LG Belardin LB Echem C Akamine EH Antoniassi MP Andretta RR. Systemic arterial hypertension leads to decreased semen quality and alterations in the testicular microcirculation in rats. Sci Rep (2019) 9(1):11047. doi: 10.1038/s41598-019-47157-w 31363128 PMC6667492

[178] Felix-Patricio B Medeiros JL Jr. De Souza DB Costa WS Sampaio FJ. Penile histomorphometrical evaluation in hypertensive rats treated with sildenafil or enalapril alone or in combination: A comparison with normotensive and untreated hypertensive rats. J Sex Med (2015) 12(1):39–47. doi: 10.1111/jsm.12750 25407323

[179] Akinyemi AJ Adedara IA Thome GR Morsch VM Rovani MT Mujica LKS. Dietary supplementation of ginger and turmeric improves reproductive function in hypertensive Male rats. Toxicol Rep (2015) 2:1357–66. doi: 10.1016/j.toxrep.2015.10.001 PMC5598100 28962478

[180] Akagashi K Itoh N Kumamoto Y Tsukamoto T Suzuki T Ohta Y. Hypertensive changes in intratesticular arteries impair spermatogenesis of the stroke-prone spontaneously hypertensive rat. J Androl (1996) 17(4):367–74. doi: 10.1002/j.1939-4640.1996.tb01802.x 8889699

[181] Militante JD Lombardini JB. Treatment of hypertension with oral taurine: Experimental and clinical studies. Amino Acids (2002) 23(4):381–93. doi: 10.1007/s00726-002-0212-0 12436205

[182] Drobnis EZ Nangia AK. Psychotropics and Male reproduction. Adv Exp Med Biol (2017) 1034:63–101. doi: 10.1007/978-3-319-69535-8_8 29256128

[183] Kaar SJ Natesan S McCutcheon R Howes OD. Antipsychotics: Mechanisms underlying clinical response and side-effects and novel treatment approaches based on pathophysiology. Neuropharmacology (2020) 172:107704. doi: 10.1016/j.neuropharm.2019.107704 31299229

[184] Dilsaver SC. Antipsychotic agents: A review. Am Fam Physician (1993) 47(1):199–204. 8418582

[185] Apter A Dickerman Z Gonen N Assa S Prager-Lewin R Kaufman H. Effect of chlorpromazine on hypothalamic-Pituitary-Gonadal function in 10 adolescent schizophrenic boys. Am J Psychiatry (1983) 140(12):1588–91. doi: 10.1176/ajp.140.12.1588 6359897

[186] Raji Y Ifabunmi SO Akinsomisoye OS Morakinyo AO Oloyo AK. Gonadal responses to antipsychotic drugs: Chlorpromazine and thioridazine reversibly suppress testicular functions in albino rats. Int J Pharmacol (2005) 1(3):287–92. doi: 10.3923/ijp.2005.287.292

[187] Hong CY Chaput de Saintonge DM Turner P. Effects of chlorpromazine and other drugs acting on the central nervous system on human sperm motility. Eur J Clin Pharmacol (1982) 22(5):413–6. doi: 10.1007/BF00542545 7117352

[188] Namyniuk MA Chaturvedi AK. Influence of chlorpromazine on motility and calcium uptake of boar sperm. Arch Int Pharmacodyn Ther (1988) 292:286–304. 2969221

[189] Ilgin S. The adverse effects of psychotropic drugs as an endocrine disrupting chemicals on the hypothalamic-pituitary regulation in Male. Life Sci (2020) 253:117704. doi: 10.1016/j.lfs.2020.117704 32339542

[190] Oyovwi MO Nwangwa EK Ben-Azu B Edesiri TP Emojevwe V Igweh JC. Taurine and coenzyme Q10 synergistically prevent and reverse chlorpromazine-induced psycho-neuroendocrine changes and cataleptic behavior in rats. Naunyn Schmiedebergs Arch Pharmacol (2021) 394(4):717–34. doi: 10.1007/s00210-020-02003-z 33146779

[191] Ghadirian AM Chouinard G Annable L. Sexual dysfunction and plasma prolactin levels in neuroleptic-treated schizophrenic outpatients. J Nerv Ment Dis (1982) 170(8):463–7. doi: 10.1097/00005053-198208000-00004 6124580

[192] Howes OD Wheeler MJ Pilowsky LS Landau S Murray RM Smith S. Sexual function and gonadal hormones in patients taking antipsychotic treatment for schizophrenia or schizoaffective disorder. J Clin Psychiatry (2007) 68(3):361–7. doi: 10.4088/jcp.v68n0302 PMC3666283 17388704

[193] Nordgren TM Bailey KL. Pulmonary health effects of agriculture. Curr Opin Pulm Med (2016) 22(2):144–9. doi: 10.1097/MCP.0000000000000247 PMC4764055 26761627

[194] Sankpal UT Pius H Khan M Shukoor MI Maliakal P Lee CM. Environmental factors in causing human cancers: Emphasis on tumorigenesis. Tumour Biol (2012) 33(5):1265–74. doi: 10.1007/s13277-012-0413-4 22614680

[195] Jubendradass R D’Cruz SC Rani SJ Mathur PP. Nonylphenol induces apoptosis *Via* mitochondria- and fas-L-Mediated pathways in the liver of adult Male rat. Regul Toxicol Pharmacol (2012) 62(3):405–11. doi: 10.1016/j.yrtph.2012.01.004 22306827

[196] Yi L Dai J Chen Y Tong Y Li Y Fu G. Reproductive toxicity of cadmium in pubertal Male rats induced by cell apoptosis. Toxicol Ind Health (2021) 37(8):469–80. doi: 10.1177/07482337211022615 34128436

[197] Meli R Monnolo A Annunziata C Pirozzi C Ferrante MC. Oxidative stress and bpa toxicity: An antioxidant approach for Male and female reproductive dysfunction. Antioxid (Basel) (2020) 9(5). doi: 10.3390/antiox9050405 PMC7278868 32397641

[198] Kim KH Kabir E Jahan SA. Exposure to pesticides and the associated human health effects. Sci Total Environ (2017) 575:525–35. doi: 10.1016/j.scitotenv.2016.09.009 27614863

[199] Sinha N Adhikari N D. KS. effect of endosulfan during fetal gonadal differentiation on spermatogenesis in rats. Environ Toxicol Pharmacol (2001) 10(1-2):29–32. doi: 10.1016/s1382-6689(01)00066-7 11382554

[200] Long H Jin Y Lin M Sun Y Zhang L Clinch C. Fluoride toxicity in the Male reproductive system. Fluoride (2009) 42(4):260–76. doi: 10.1093/eurpub/ckp126

[201] Sun Z Niu R Wang B Wang J. Altered sperm chromatin structure in mice exposed to sodium fluoride through drinking water. Environ Toxicol (2014) 29(6):690–6. doi: 10.1002/tox.21796 22865829

[202] Abdel Moneim AE. Prevention of carbon tetrachloride (Ccl4)-induced toxicity in testes of rats treated with physalis peruviana l. Fruit Toxicol Ind Health (2016) 32(6):1064–73. doi: 10.1177/0748233714545502 25147302

[203] Pizent A Tariba B Zivkovic T. Reproductive toxicity of metals in men. Arh Hig Rada Toksikol (2012) 63 Suppl 1:35–46. doi: 10.2478/10004-1254-63-2012-2151 22548851

[204] Lafuente A Marquez N Perez-Lorenzo M Pazo D Esquifino AI. Pubertal and postpubertal cadmium exposure differentially affects the hypothalamic-Pituitary-Testicular axis function in the rat. Food Chem Toxicol (2000) 38(10):913–23. doi: 10.1016/s0278-6915(00)00077-6 11039325

[205] Guo C Huang C Chen S Wang Hsu G. Serum and testicular testosterone and nitric oxide products in aluminum-treated mice. Environ Toxicol Pharmacol (2001) 10(1-2):53–60. doi: 10.1016/s1382-6689(01)00069-2 11382556

[206] Zubair M Ahmad M Qureshi ZI. Review on arsenic-induced toxicity in Male reproductive system and its amelioration. Andrologia (2017) 49(9). doi: 10.1111/and.12791 28133775

[207] Chang SI Jin B Youn P Park C Park JD Ryu DY. Arsenic-induced toxicity and the protective role of ascorbic acid in mouse testis. Toxicol Appl Pharmacol (2007) 218(2):196–203. doi: 10.1016/j.taap.2006.11.009 17188728

[208] Ryan JL. Ionizing radiation: The good, the bad, and the ugly. J Invest Dermatol (2012) 132(3 Pt 2):985–93. doi: 10.1038/jid.2011.411 PMC3779131 22217743

[209] Herrera Ortiz AF Fernandez Beaujon LJ Garcia Villamizar SY Fonseca Lopez FF. Magnetic resonance versus computed tomography for the detection of retroperitoneal lymph node metastasis due to testicular cancer: A systematic literature review. Eur J Radiol Open (2021) 8:100372. doi: 10.1016/j.ejro.2021.100372 34458506 PMC8377546

[210] Kamiguchi Y Tateno H Mikamo K. Dose-response relationship for the induction of structural chromosome aberrations in human spermatozoa after in vitro exposure to tritium beta-rays. Mutat Res (1990) 228(2):125–31. doi: 10.1016/0027-5107(90)90068-f 2300065

[211] Zhou DD Hao JL Guo KM Lu CW Liu XD. Sperm quality and DNA damage in men from jilin province, China, who are occupationally exposed to ionizing radiation. Genet Mol Res (2016) 15(1). doi: 10.4238/gmr.15018078 27050976

[212] Moller AP Mousseau TA Lynn C Ostermiller S Rudolfsen G. Impaired swimming behaviour and morphology of sperm from barn swallows hirundo rustica in Chernobyl. Mutat Res (2008) 650(2):210–6. doi: 10.1016/j.mrgentox.2007.12.006 18218334

[213] Gong EJ Shin IS Son TG Yang K Heo K Kim JS. Low-Dose-Rate radiation exposure leads to testicular damage with decreases in Dnmt1 and Hdac1 in the murine testis. J Radiat Res (2014) 55(1):54–60. doi: 10.1093/jrr/rrt090 24027299 PMC3885123

