# Supplementary material for: The role of taurine in male reproduction: Physiology, pathology and toxicology
Source: Front Endocrinol (Lausanne). 2023 Jan 18;14:1017886. doi: 10.3389/fendo.2023.1017886 (PMC9889556; doi:10.3389/fendo.2023.1017886)
Supplement: Supplementary file 1 [file DataSheet_1.docx]

Supplementary Material

## Supplementary Figures


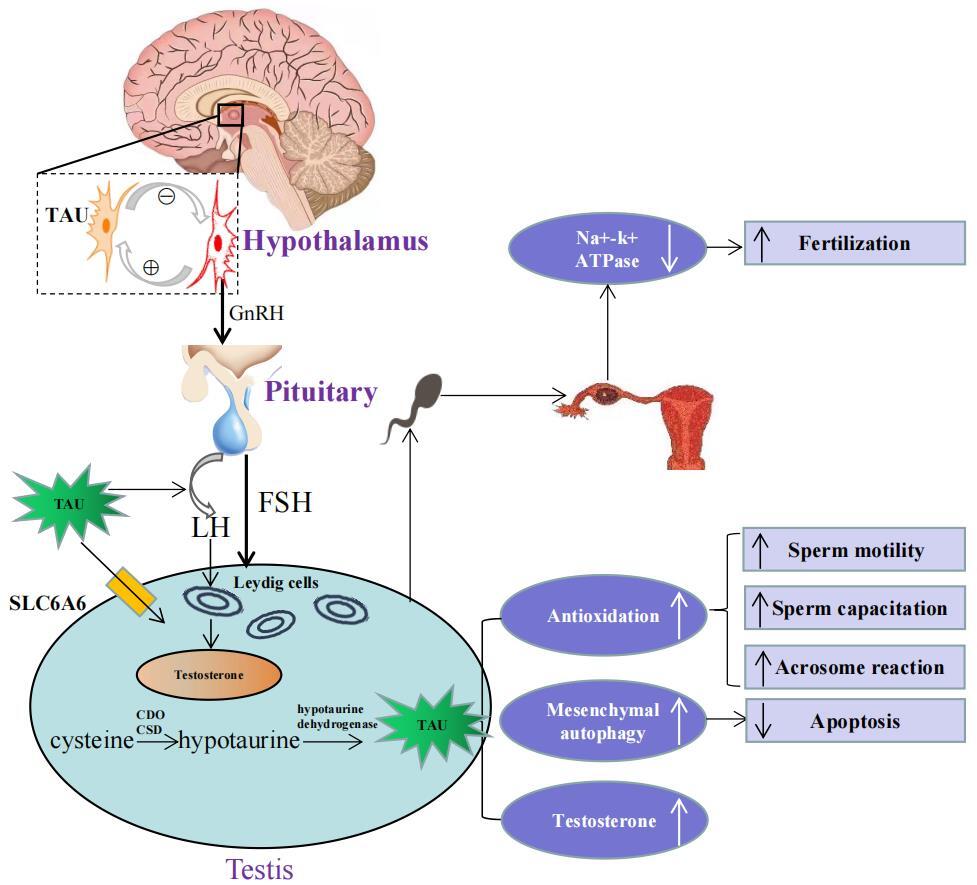


**Supplementary Figure 1.** Schematic illustration of physiological role of taurine in hypothalamus-pituitary-testis endocrine axis. Top left portion shows the positive and negative feedback between taurine-secreting neurons and GnRH-secreting neurons in the hypothalamus. Lower left portion indicates that taurine regulates the secretion of FSH and LH by directly acting on the pituitary, and then LH acts on Leydig cells to promote testosterone secretion. Moreover, taurine can be transported to testis through transporter SLC6A6, and can also be synthesized endogenously in testis. Lower right portion indicates that the physiological mechanism and effects of taurine in testis, including: (i) enhancing antioxidation to promote spermatogenesis, sperm capacitation and acrosome reaction; (ii) enhancing the autophagy process of interstitial cells to inhibit cell apoptosis; (iii) promoting the secretion of testosterone. Top right portion shows in the fallopian tube, taurine reduced cellular K^+^ influx by inhibiting Na^+^–K^+^ ATPase activity, thereby avoiding high K^+^ inhibition of sperm motility to promote conception. TAU, taurine; SLC6A6, Sodium-and chloride-dependent taurine transporter; CDO, cysteine dioxygenase; CSD, cysteine sulfinate decarboxylase; GnRH, gonadotropin-releasing
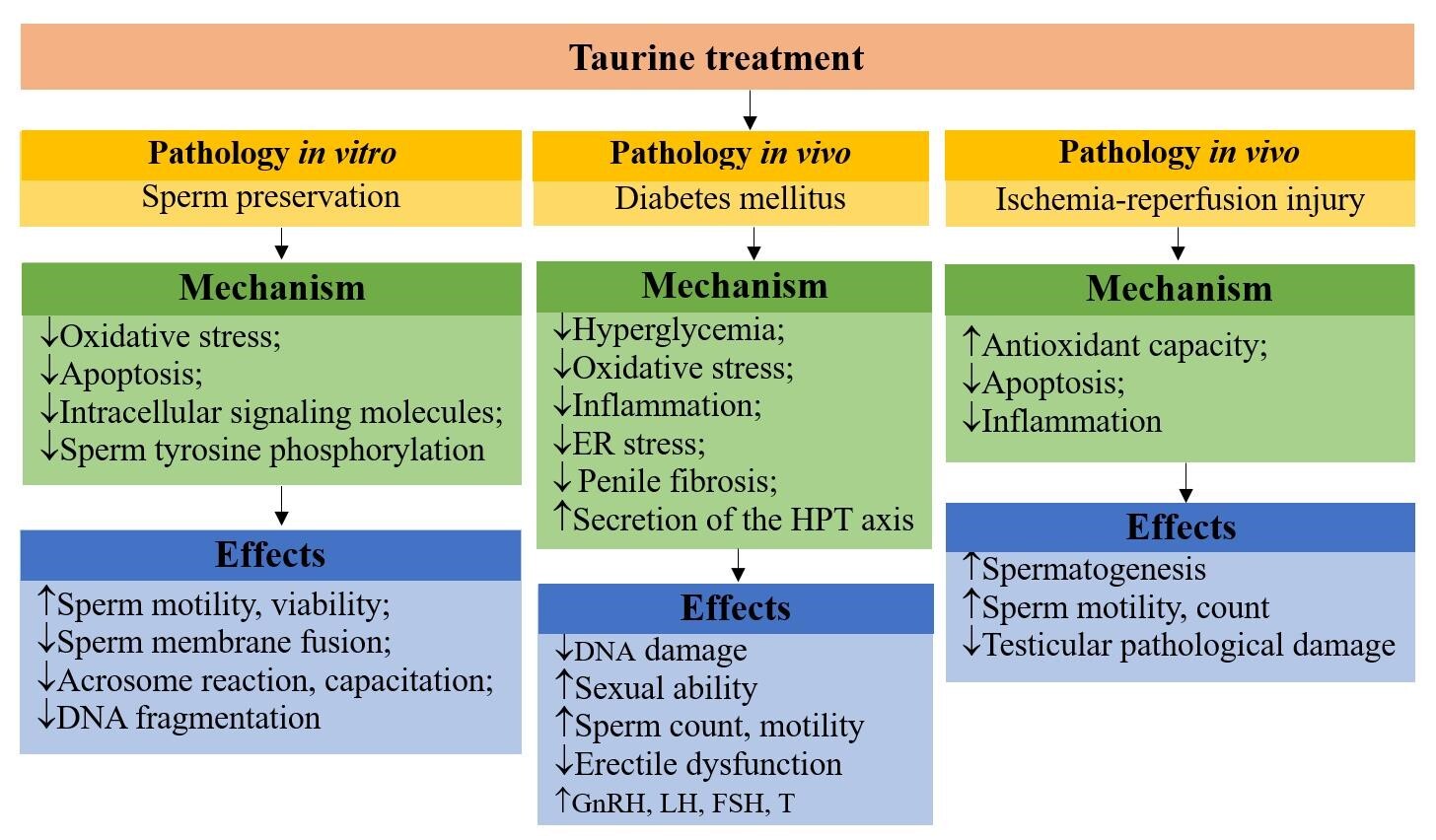
hormone; FSH, follicle-stimulating hormone; LH, luteinizing hormone.

**Supplementary Figure2(A)**: schematic mechanisms of taurine protecting male reproductive function in pathologies. ↑indicates increase; ↓indicates decrease. Firstly, in sperm preservation in vitro, taurine improved sperm motility, viability and reduced membrane fusion, acrosome and capacitation during preservation by reducing oxidative stress (eliminating excessive ROS, MDA and LOPs, increasing the activity of antioxidant enzymes SOD, CAT and MMP), inhibiting sperm apoptosis (reducing DNA fragmentation), reducing intracellular signal molecules (Ca^2+^, cAMP and DAG) and reducing sperm protein tyrosine phosphorylation levels. Secondly, in the male reproductive dysfunction caused by diabetes, taurine improved sperm count and motility, enhanced sexual response and sexual ability, and alleviated erectile dysfunction by reducing hyperglycemia, oxidative stress (eliminating excessive ROS and LOPs, increasing the activities of antioxidant enzymes SOD, CAT and GPx) anti-inflammation (reducing pro-inflammatory cytokines TNF-α and IL-6), inhibiting ER stress and penile fibrosis, and promoting the secretion of HPT axis. Thirdly, in terms of testicular ischemia-reperfusion injury, taurine increased sperm count, motility and spermatogenesis, and decreased the pathological damage of testicular tissue by enhancing antioxidant capacity (enhancing the activity of antioxidant enzyme SOD, eliminating excessive ROS and LOPs), inhibiting apoptosis (reducing NO level and eNOS expression) and inhibiting inflammatory reaction (reducing neutrophil recruitment). GnRH: Gonadotrophin-releasing hormone, LH, Luteinizing hormone, FSH: Follicle-stimulating hormone, ER stress: Endoplasmic reticulum stress, HPT axis: Hypothalamic-pituitary-thyroid.


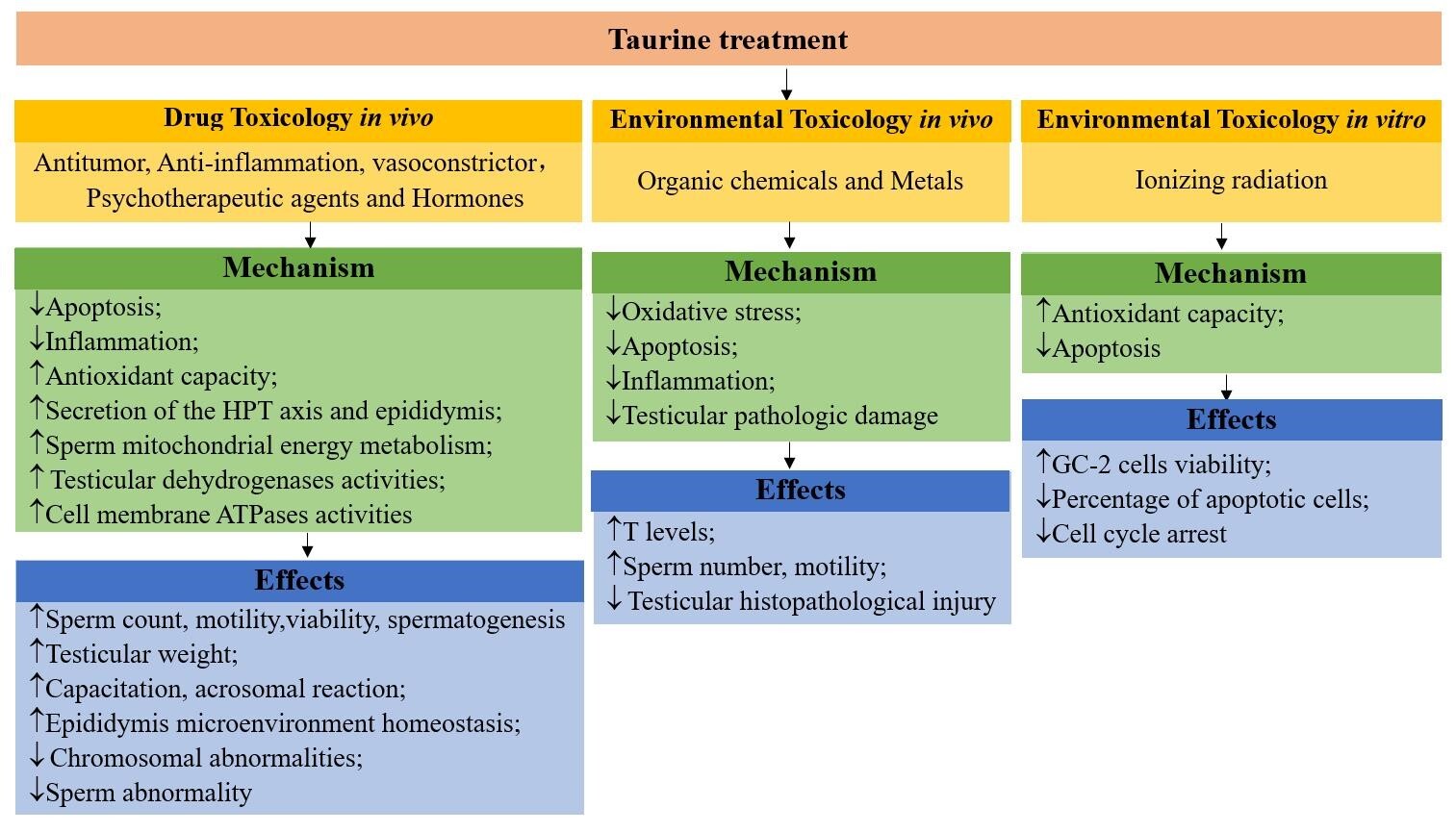


**Supplementary Figure2(B)**: schematic mechanisms of taurine protecting male reproductive function in toxicology.↑indicates increase;↓indicates decrease. Firstly, in terms of drug-induced male reproductive dysfunction, taurine improved sperm count, motility, motility and testicular weight, and also enhanced spermatogenesis, acrosome and capacitation, while maintaining epididymis microenvironment homeostasis and reducing chromosomal abnormalities by increasing antioxidant activity (increasing GSH and antioxidant enzyme SOD, CAT activity; reducing H2O2, ROS, MDA), inhibiting apoptosis (reducing cytochrome c and caspase-3), reducing inflammation (reducing TNF-α, ICAM-1 and MMP-9), improving sperm mitochondria energy metabolism, promoting epididymis secretion (carnitine, SA, α-Glu and ACP) and HPT axis secretion activity (LH, FSH and T), enhancing testicular dehydrogenase (3β-HSD, 17β-HSD, G6PDH, LDH-X) and electrogenic pump (Na^+^/K^+^, Ca^2+^, Mg^2+^, H^+^-ATPase) activity. Secondly, in terms of environmental toxins, taurine increased sperm count, motility, and testosterone levels, and reduced pathological damage to testicular tissue by reducing oxidative stress (increasing GSH levels and antioxidant enzymes GPx, GST, CAT, SOD Activity, reducing ROS, MDA), apoptosis (reducing Bax protein expression), inflammatory response (reducing NO concentration), testicular histopathological changes (reducing seminiferous tubule atrophy and cell degeneration). Thirdly, in terms of ionizing radiation, taurine increased the viability and cycle of GC-2 cells by increasing antioxidant capacity (increasing the expression of Nrf 2 and HO-1) and inhibiting apoptosis (inhibiting the activation of Fas/FasL pathway). T: testosterone.
